# A spatial database of CO_2_ emissions, urban form fragmentation and city-scale effect related impact factors for the low carbon urban system in Jinjiang city, China

**DOI:** 10.1016/j.dib.2020.105274

**Published:** 2020-02-11

**Authors:** Shaoqing Dai, Shudi Zuo, Yin Ren

**Affiliations:** a Key Laboratory of Urban Environment and Health, Institute of Urban Environment, Chinese Academy of Sciences, Xiamen, 361021, China; b University of Chinese Academy of Sciences, Xiamen, 361021, China; c Ningbo Urban Environment Observation and Research Station-NUEORS, Chinese Academy of Sciences, Ningbo, 315800, China

**Keywords:** Urban form, CO_2_ emissions gridded maps, GIS, Landscape ecology

## Abstract

This paper presented the spatial database collected in 2013 for mitigating the urban carbon emissions of Jinjiang city, China. The database included the high-resolution CO_2_ emissions gridded maps, urban form fragmentation evaluation maps, and city-scale effect related impact factors distribution maps at 30 m and 500 m. We collected the multi-sources data including statistical, vector, and raster data from open-access websites and local governments. We used a general hybrid approach based on global downscaled and bottom-up elements to produce the CO_2_ emissions gridded maps. The urban fragmentation was measured by the landscape fragmentation metrics under the feature scale and the accurate identification of the urban functional districts. The percentage of the urban area and the points of interest (POI) density representing the city-scale effect related impact factors were calculated in each grid by the land use and POI data. Our database could be used for the validation of urban CO_2_ emissions estimation at the city scale. The landscape metrics and city-scale effect related impact factors maps can also be used for evaluating the socio-economic status in order to solve the other urban spatial planning problems.

**Table:** Specifications Table

Subject	Environmental Sciences
Specific subject area	Landscape ecology, Climate change, Carbon emissions
Type of data	Text and Geo-tiff raster
How data were acquired	The raw data used to produce the CO_2_ emissions gridded maps were from the statistical yearbooks (energy consumption by sectors), public remote sensing products (NPP-VIIRS, DEM) and the Jinjiang municipal government department (population, land use and road network). Besides, the other raw data used to identify the urban landscape functional districts were obtained from the Jinjiang municipal government department (city master plan) and the Baidu map.com (points of the interest data). The analyzed data were produced by authors using data fusion and calculation based on GIS and RS technologies through the raw data.
Data format	Raw and Analyzed
Parameters for data collection	Raw spatial data included the vector master planning (2010–2030), the vector land use data, point of interest data which were uniformed into 16 urban functional subtypes, road network data with 5 different road levels, digital elevation model, and nightlight imagery. Excepting for them, the population per block, the GDP, energy consumption and related socio-economic factors data were collected and calculated as well. All the data were collected for the year of 2013.
Description of data collection	Raw data were collected from a number of major sources including: NOAA website (https://ngdc.noaa.gov/eog/viirs/index.html), 2014 Quanzhou statistical yearbook, IPCC report, 2014 China Urban Statistical Yearbook, 2014 China Urban Construction Yearbook, 2013 Jinjiang National Economic and Social Development Statistical Bulletin, Geospatial Data Cloud website (http://www.gscloud.cn/), Planning Bureau of Jinjiang, Public Security Bureau of Jinjiang, and Baidu.com.
Data source location	Institution: Institute of Urban Environment, Chinese Academy of Sciences City/Town/Region: Jinjiang Country: China
Experimental factors	The project coordinate system of the spatial data was the Albers WGS 1984. The data fusion and analysis were run out with Arcmap software.
Data accessibility	Data identification number: 10.5281/zenodo.3566072. Direct URL to the analyzed dataset: http://doi.org/10.5281/zenodo.3566072.
Related research article	Authors' names: Shudi Zuo, Shaoqing Dai, Yin Ren. Title: More fragmentized urban form more CO_2_ emissions? A comprehensive relationship from the combination analysis across different scales Journal: Journal of Cleaner Production https://doi.org/10.1016/j.jclepro.2019.118659

**Table:** 

**Value of the Data** • The provided dataset of gridded CO_2_ emissions could be used for the validation of other CO_2_ emissions studies at different resolution scales. • The urban functional district/zone maps could be used to optimize the urban form and design a carbon emissions mitigation strategy. • The city-scale effect impact factors could be used to evaluate the socio-economic status of this city.

## 1 Data

**Fig. 1:**
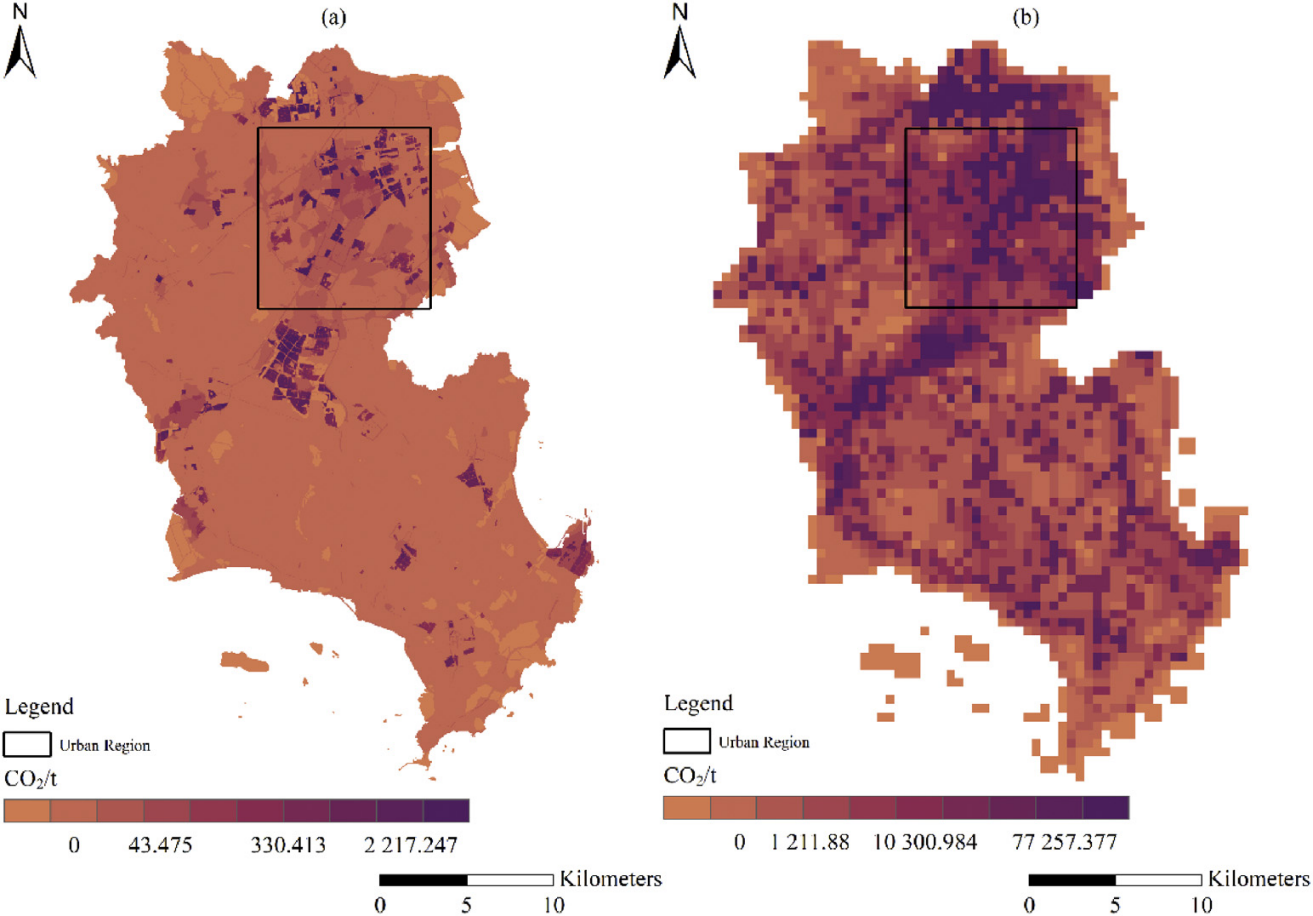
The high-resolution CO_2_ girded maps. (a) and (b) represent the CO_2_ emissions gridded maps at R_30m_ and R_500__m_ respectively.

**Fig. 2:**
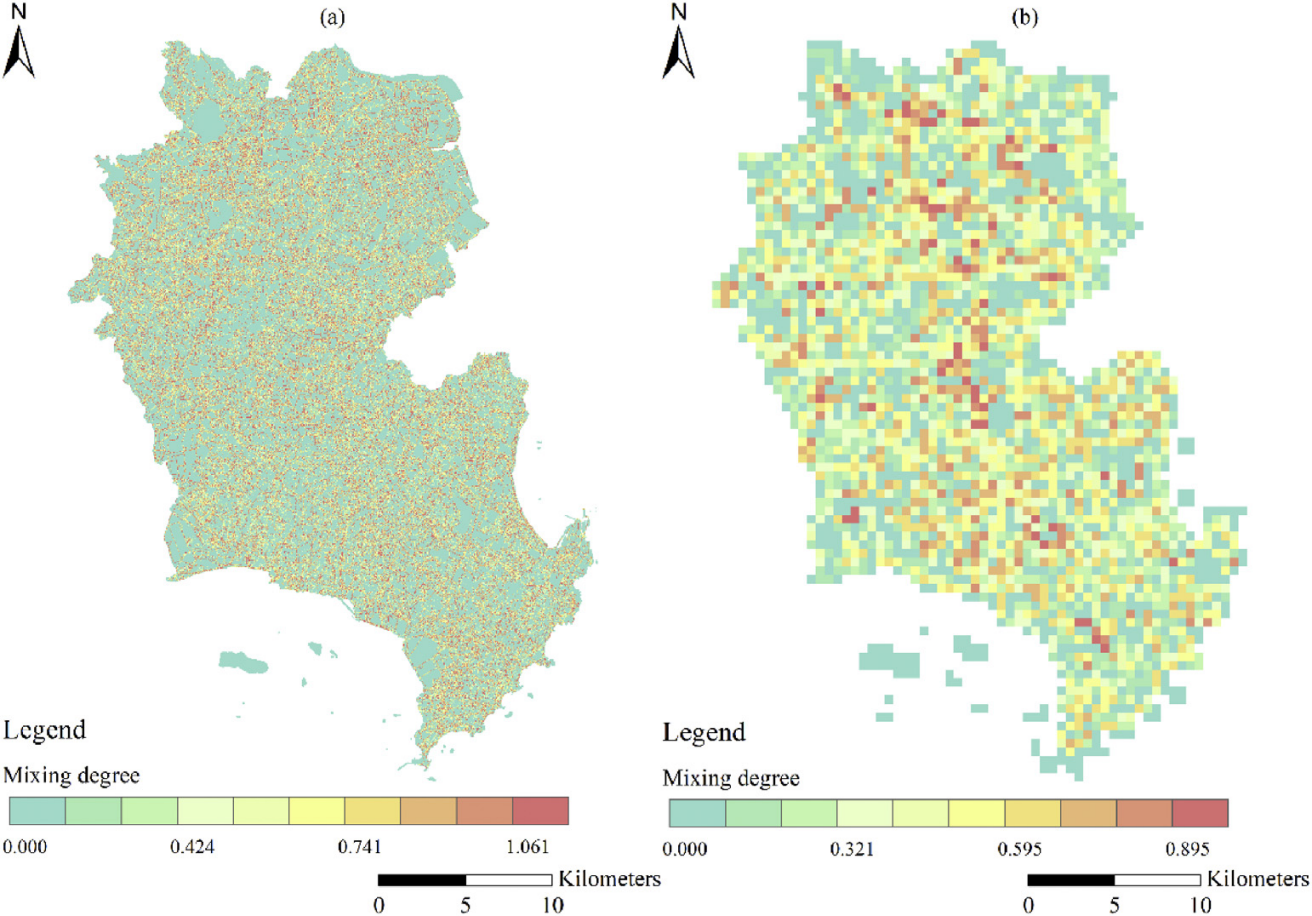
The mixing degree of urban functional district (UFD). (a) and (b) represent the mixing degree of UFD at R_30m_ and R_500m_ respectively.

**Fig. 3:**
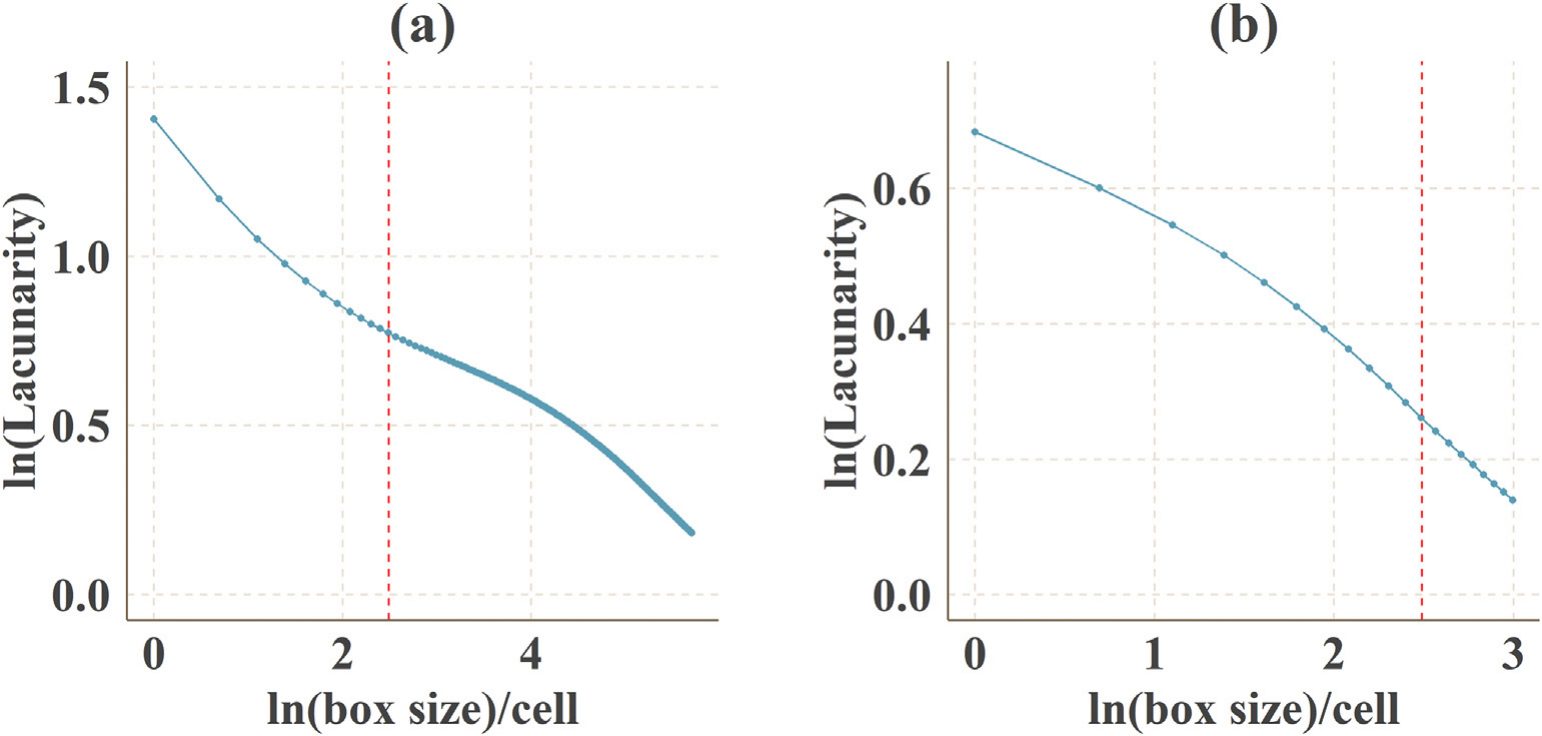
Log-log plots of Lacunarity index versus sliding frame size. (a) and (b) represented R_30 m_ and R_500 m_ respectively. The inflection points were indicated by the dotted red line.

**Fig. 4:**
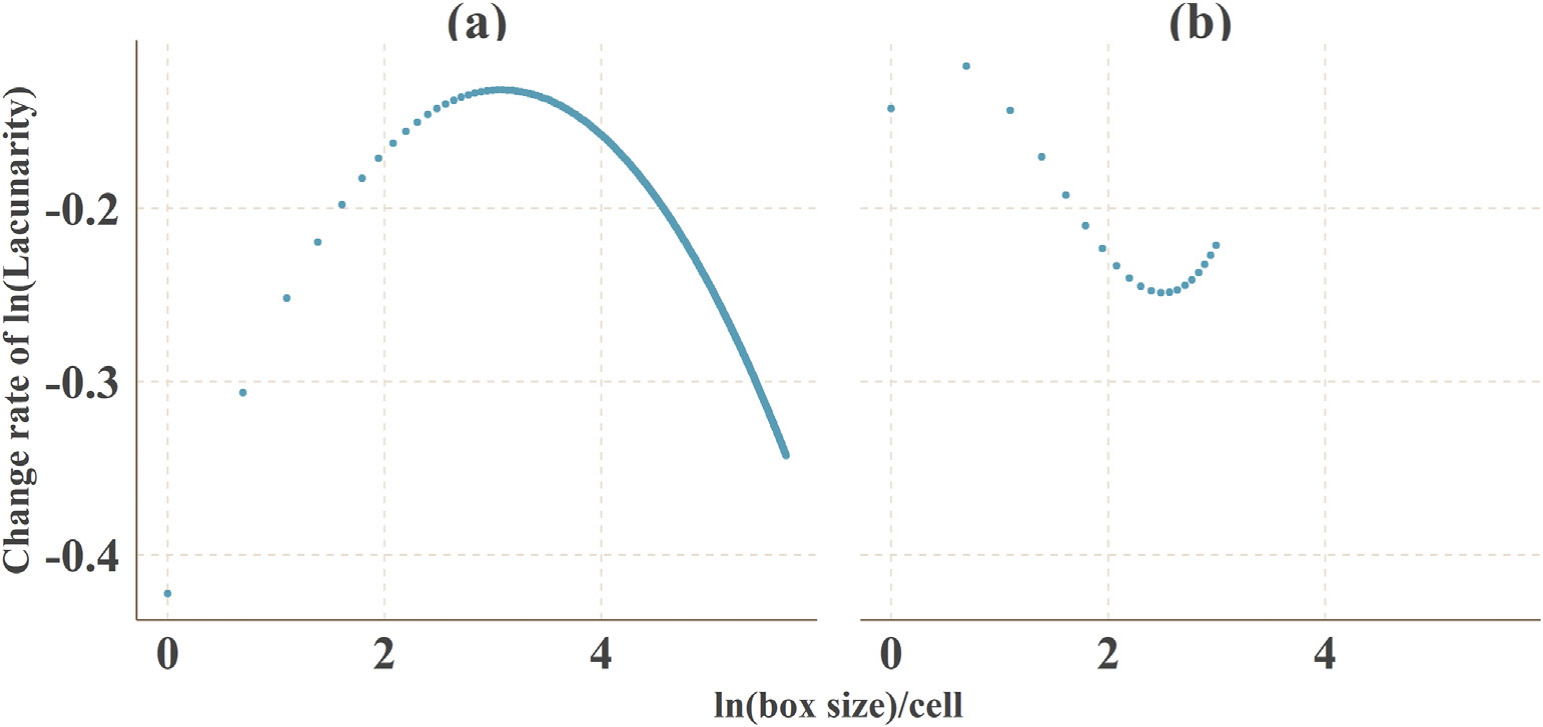
The differential of fitting polynomial for log-log curve of size-Lacunarity index for the feature scale. (a) and (b) represented R_30 m_ and R_500 m_ respectively.

**Fig. 5:**
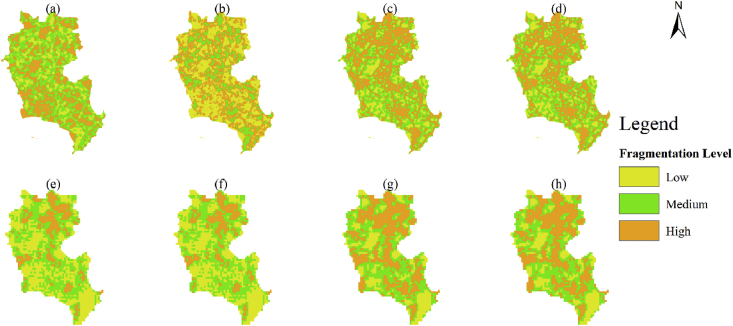
Fragmentation level based on the landscape metrics. a, b, c, and d are at R_30m_. e, f, g, and h are at R_500m_. (a) and (e) are NP, (b) and (f) are PD, (c) and (g) are DIVISION, (d) and (h) are MESH.

**Fig. 6:**
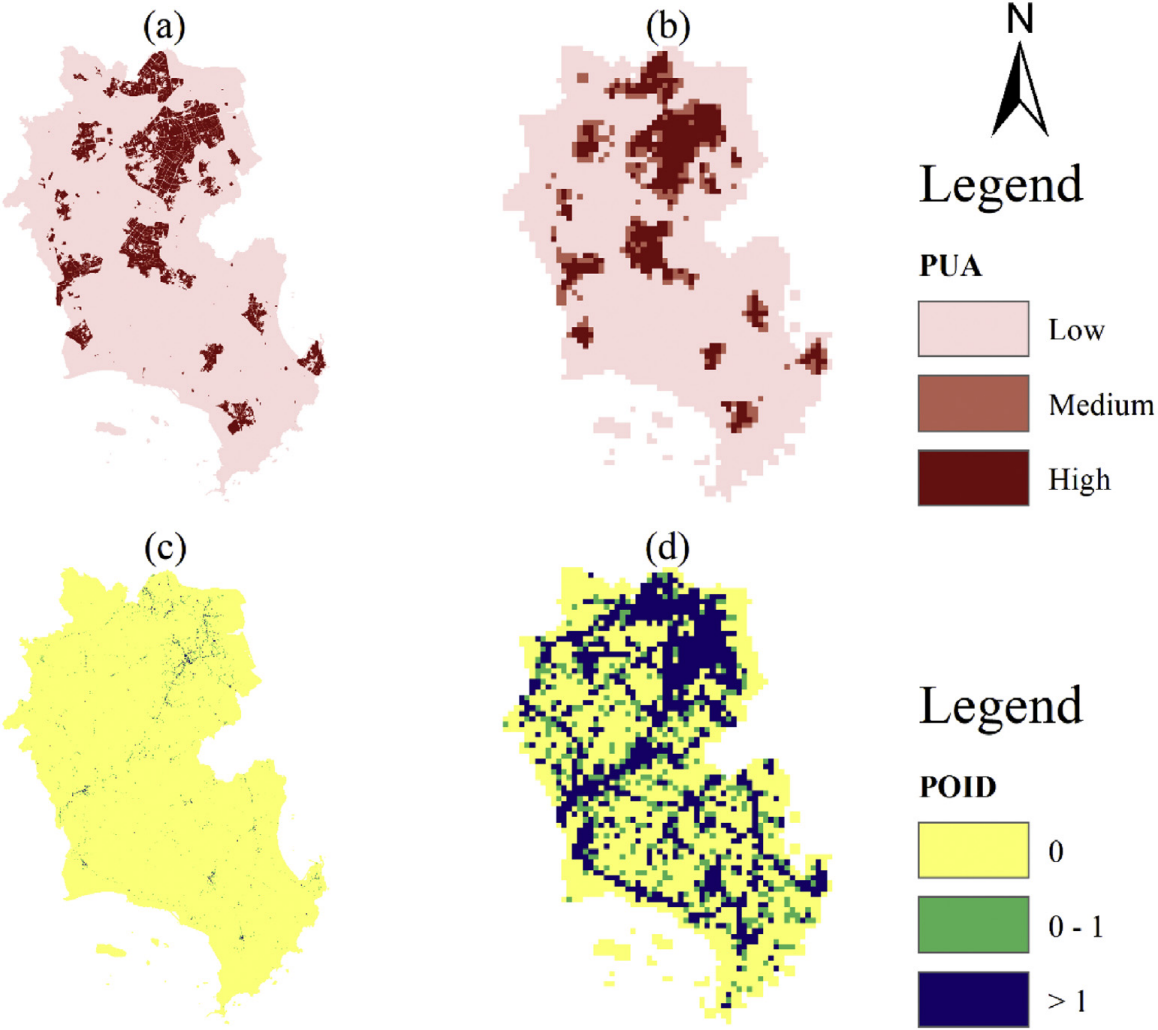
PUA and POID at different resolutions. (a) and (c) are at R_30m_, (b) and (d) are at R_500m_.

A spatial database of low carbon urban system represented the spatial distribution maps of CO_2_ emissions, urban form metrics (urban landscape fragmentation), proportion of urban area (PUA) and points of interest density (POID) at two resolutions (30 m (R_30m_) and 500 m (R_500m_)) in 2013 Jinjiang city, China. The data were produced from ArcGIS 10.2, Apack 2.23, Fragstats 4.2 and R 3.5.3. In order to produce the spatial database, 10 types of raw data (Table 1) were preprocessed into the uniformed geographical coordinate system and some of them which were used to calculate the urban form metrics were uniformed into 16 urban functional subtypes by the specific standards (Table 2, Table 3). The high-resolution CO_2_ emissions gridded maps contained the emissions from the residential, industrial and transport sectors at two resolutions (Fig. 1). The landscape mixing degree of urban functional districts were classified through the functional district types at two resolutions (Fig. 2). The fragmentation levels at two resolutions were identified by the landscape metrics (Fig. 5) which were calculated at the feature scales (Fig. 3, Fig. 4, Table 4). The spatial distribution of PUA and POID in the spatial database represented the city-scale effect impact factors (Fig. 6). The description of all the data could be seen in the Datadescription.txt.

**Table 1:** The description of raw data sources for spatial database.

Raw data	Sources	Data type
Nightlight imagery from NASA/NOAA Suomi National Polar-orbiting Partnership (NPP-VIIRS) at 500 m	https://ngdc.noaa.gov/eog/viirs/index.html	Raster
The town-level population of Jinjiang, including the number of households, urban population, non-urban population and total population distributed over the 389 blocks, villages and towns.	Public Security Bureau of Jinjiang.	Vector
Value increased of industrial GDP (10 thousand yuan), energy consumption per industrial increased value (standard coal/10 thousand yuan), emission factors of standard coal (2.773).	2014 Quanzhou statistical yearbook, IPCC report.	Statistical
Electricity, liquefied gas, coal gas and natural gas consumption of residents in Jinjiang, the heat value of liquefied gas, coal gas and natural gas, the emission factor of power grid (0.8095 t CO_2_ ·Mwh^−1^)	2014 China Urban Statistical Yearbook and 2014 China Urban Construction Yearbook, IPCC Report.	Statistical
Energy consumption of private and public (bus and cab) transportation	2013 Jinjiang National Economic and Social Development Statistical Bulletin	Statistical
DEM of Jinjiang at 30 m	http://www.gscloud.cn/	Raster
Land use map of Jinjiang in 2013 at the parcel level	Planning Bureau of Jinjiang	Vector
Master planning spatial data for Jinjiang 2010–2030 at the parcel level	Planning Bureau of Jinjiang	Vector
Baidu POI data	Baidu Map	Vector
Baidu road network	Baidu Map	Vector

**Table 2:** The functional district and their descriptions.

Functional district	Detail
Roads	All the transportation facilities in urban areas, for example, trunk road, expressway, secondary-trunk road, junctions, bus stations and so on
Industry	The production workshop, warehouse and its auxiliary facilities in industrial and mining enterprises
Water	Rivers, lakes, reservoirs, ponds, coastal waters, inland beaches, ditches with water construction, glacier, and permanent snow
Administration and Public Services	Administrative, cultural, educational, sports, health, and other facilities
Greenland and Plazas	Public places such as parks, green space, squares and so on
Commercial and Service Facilities	Business, commercial, entertainment, and other facilities
Municipal Utilities	Supply, environment, safety and other facilities
Residential	Residence and its corresponding facilities
Mixed Function	More than three different functional districts
Cropland and Orchard	Cropland, orchard, forest, grassland, agricultural facilities, rural roads and other kinds of land
Other Non-construction	Idle land, agricultural land, facilities, raised path, saline soil, swamp, sandy land, bare land
Countryside	Construction for rural residential areas
Transportation System	Railway, highway, airport, port, pipeline and so on
Special Purpose	Land of special purposes
Mining	Mining, quarrying, sand mining, salt, ground brick kiln production land, and tailings dumps
Logistics Warehousing	Material reserves, transit, distribution and other kinds of land

**Table 3:** Rules of reclassified POI into different functional districts.

Functional district	Types of POI
Commercial and Service Facilities	Hotel, restaurant, supermarket, building, bank, other types
Administration and Public Services	School, drugstores, hospital, government, other types
Roads	Parking lot
Greenland and Plazas	Parks
Transportation System	Toll station, other types
Municipal Utilities	Other types
Residential	Other types
Industry	Other types
Logistics Warehousing	Other types

**Table 4:** Fitting and inflection point of log-log curve of size-Lacunarity.

Landscape type	Fitting curve	R^2^	Inflection point
Functional district patches (30 m)	$\text{y}=-0.422\text{x}+0.094{\text{x}}^{2}-0.010{\text{x}}^{3}+1.406$	0.999	3.080
Functional district patches (500 m)	$\text{y}=-0.143\text{x}+0.061{\text{x}}^{2}-0.049{\text{x}}^{3}+0.008{\text{x}}^{4}+0.684$	0.999	0.693

## 2 Experimental design, materials and methods

### 2.1 Raw data collection and preprocess

Our methodology developed the spatial database of CO_2_ emissions, urban form fragmentation, and city-scale related impact factors (PUA and POID) at two resolutions (R_30m_ and R_500m_). All the raw data were collected to produce the gridded maps of the spatial database. The raw data included the vector master planning spatial data for Jinjiang 2010–2030, the vector land use data for Jinjiang 2013, point of interest (POI) data, road network data, the population per town in 2013, the GDP, energy consumption and related socio-economic factors in 2013, digital elevation model (DEM) at 30 m, and nightlight imagery at 500 m in 2013. The detailed information of raw data sources is shown in Table 1.

We must preprocess the spatial raw data from different sources into the specific and standard input data before developing the spatial database. For example, the master planning spatial data of Jinjiang 2010–2030, land use map of Jinjiang, and Baidu POI data have 49 functional subtypes, 31 land use subtypes, and 10 POI subtypes respectively. We aggregated the master planning spatial data and land use map into 16 urban functional districts according to the “Current land use classification standard” (GB/T 21010-2017). Detailed aggregated information could be seen in Table 2. We reclassified the Baidu POI data into the 16 specific urban functional districts by the name of POI data. Detailed reclassification information could see Table 3. Then, we used simple correction method to adjust the nightlight imagery of NPP-VIIRS, which unified the negative value into 0 and resampled it to the 500-m resolution to correct the data. Then, the Kriging spatial interpolation method was used to downscale the 500 m nightlight imagery to 30 m [1]. Besides, 30 m DEM was resampled to 500 m. So far, all the raster data mentioned in Table 1 had the images at two resolutions. Finally, all the vector and raster were unified into the WGS Albers 1984 projection coordinate system.

### 2.2 High-resolution CO_2_ gridded maps

Our study follows the 2006 IPCC guidelines for National Greenhouse Gas Inventories [2]. We produced gridded maps of CO_2_ emissions with sizes of 30 x 30 m and 500 x 500 m—resolutions which are typical in urban studies—based on multi-source geospatial CO_2_ emissions data.

In this study, gridded maps of CO_2_ emissions were constructed using a general hybrid approach based on global downscaled and bottom-up elements (e.g., industrial area). The total CO_2_ emissions in each grid were calculated as follows:

(1) $$Grid_{i}\;=\;\sum_{l=1}^{3}C_{l}\cdot Weight_{i,l}=\sum_{l=1}^{3}AL_{l}\cdot EF_{l}\cdot Weight_{i,l}$$

where *Grid*_*i*_ is the total CO_2_ emissions (unit: *t*) at the *i*^th^ grid (*i* = *1*, *2*, *3*…, *n*), *C*_*l*_ (units: *t*) is the total amount of CO_2_ emissions from different emission sources, *AL*_*l*_ (units: t) is the total energy consumption from different emission sources, and EF_l_ (units: t/t CO_2_) is the emission factor for different emission sources based on the IPCC method [2] at the *i*^th^ grid (*l* = *p*, *I*, *T*, which represent residential, industrial, and transport emissions, respectively), and *Weight*_*i,l*_ is the weight of the specific emission type on-grid *i*. In fact, *Weight*_*i,l*_ is the mathematical form of spatial proxies.

As formula (1) showed, we calculated the total CO_2_ emissions based on energy consumption values within the urban geographic boundary of Jinjiang City in 2013 firstly. The total CO_2_ emissions could be divided into three sectors: residential, industrial, and transport emissions.

The equation for residential emissions was as follows:

(2) $$C_{1}=E_{1}\times EF_{1i}/P$$

(3) $$C_{2}=F_{2}\times NVI_{1}\times EF_{2}/P+F_{3}\times NVI_{2}\times EF_{3}\times M_{1}/P+F_{4}\times NVI_{3}\times EF_{4}\times M_{2}/P$$

(4) $${\text{C}}_{p}=C_{1}+C_{2}$$

where *C*_*1*_ was the electricity CO_2_ emission per capita; *E*_*1*_ was the household electricity consumption; *EF*_*1i*_ was the carbon emission factor of the power grid, which was 0.8095 tCO_2_·Mwh^−1^ (Fujian Province belongs to the East China regional power grid); *P* was the population; *C*_*2*_ was the gas CO_2_ emissions per capita; *F*_*2*_, *F*_*3*_ and *F*_*4*_ were the quantities of household liquefied gas, gas and natural gas consumption; *NVI*_*1*_ (50.179 MJ·kg^−1^), *NVI*_*2*_ (38.931 MJ·m^−3^) and *NVI*_*3*_ (38.7 MJ·kg^−1^) were the heating values of liquefied gas, gas and natural gas, respectively; *EF*_*2*_ (0.06307 kgCO_2_·MJ^−1^), *EF*_*3*_ (0.0561 kgCO_2_·MJ^−1^) and *EF*_*4*_ (0.0444 kgCO_2_·MJ^−1^) were the carbon emission factors of liquefied gas, gas and natural gas, respectively; *M*_*1*_ (0.45 kg·m^−3^) and *M*_*2*_ (0.717 kg·m^−3^) were the density of gas and natural gas; *C*_*p*_ was the mean emissions per capita of CO_2_.

The equation for industrial emission was as follows:

(5) $$C_{I}=k_{1}\times I_{growth}\times K_{I}$$

where *C*_*I*_ was the industrial emission; *K*_*I*_ was the energy consumption of industrial enterprises above a designated size (t standard coal per million yuan); *I*_*growth*_ was the increment of the industrial enterprises (million yuan); *k*_*1*_ was the standard coal CO_2_ emission factor in the city, which was 2.773 t CO_2_ per t standard coal. The standard coal was calculated from the energy consumed during production process that generated greenhouse gases, (e.g. cement and lime production). Besides, energy consumption per industrial increased value was equal to the industrial energy consumption divided by the increased value of industrial GDP, details can be seen at http://www.stats.gov.cn/tjsj/tjgb/qttjgb/qgqttjgb/201007/t20100715_30644.html.

The equation for transportemissions was as follows:

(6) $$C_{T}=Q_{1}L_{1}\lambda_{1}k_{2}+Q_{2}L_{2}\lambda_{2}k_{2}+Q_{3}L_{3}\lambda_{3}k_{2}+T_{1}K_{T1}k_{1}+T_{2}K_{T2}k_{1}$$

where *C*_*T*_ was the transport CO_2_ emissions; *Q*_*1*_, *Q*_*2*_ and *Q*_*3*_ were the numbers of buses, taxis and private cars, respectively; *L_1_*, *L_2_*, and *L_3_* were the annual total mileages of buses, taxis and private cars, respectively; *λ_1_* (32 L·km^−1^), *λ*_*2*_ (10 L·km^−1^) and *λ*_*3*_ (10 L·km^−1^) were the oil consumption factors per hundred kilometers, respectively; *T*_*1*_ and *T*_*2*_ were passenger and freight turnover, respectively; *K*_*T1*_ (11.6 kg standard coal per thousand person kilometers) and *K*_*T2*_ (1.9 kg standard coal per hundred tons kilometers) were the units of energy consumption of the passenger transport and freight; *k*_*1*_ (2.773 t CO_2_ per t standard coal) was the CO_2_ emission coefficients of standard coal and *k*_*2*_ (2.314 kg·L^−1^) was the gasoline CO_2_ emission coefficient. The mileage of buses (70 080 km·year^−1^), taxis (12 000 km·year^−1^) and private cars (20 000 km·year-1) was calculated in terms of references [[3], [4], [5], [6]].

To produce gridded maps of CO_2_ emissions, we constructed spatial proxies that reflected the distribution of CO_2_ emissions and allocated the total emissions of certain regions to each grid according to different weights. The spatial proxies were the high-resolution gridded population map, industrial land map, maps of nighttime light intensity, and the areas of various types of the road which were generated from multi-source data including digital elevation models, nighttime light imagery from the NPP-VIIRS, land use map, POIs and road network map. In order to produce the maps at two spatial resolutions, the spatial proxies were both generated using geospatial data at two resolutions. For instance, the high accuracy population map at 500 m relied on the digital number (DN) value of nighttime light imagery from NPP-VIIRS. However, more proxy variables should be added for the OLS regression models at 30 m such as the elevation and the area of different land-use subtypes. The product of the nighttime light intensity and the industrial land map were used to generate the industrial proxies at two resolutions. Road areas were calculated based on the national road construction standard which states that different classes of road have different road widths at two resolutions [7]. Road areas representing the transport emissions were calculated by multiplying road widths and road lengths in each grid cell using the ArcGIS 10.2 software. Finally, we generate the CO_2_ emission gridded maps by overlaying these proxies at two resolutions (Fig. 1).

High-resolution CO_2_ emissions gridded maps were obtained by combining global downscaled and bottom-up approaches with spatial analysis models; however, some uncertainties of input variables existed. The uncertainties of input variables were propogated and mapped when obtaining the high-resolution CO_2_ emission map. The uncertainties should be explained and analyzed [8].

The first step was to analyze the input variables, the output variables and the whole distribution model. The input variables included total CO_2_ estimate and spatial proxies, as shown in formula (1). As to the CO_2_ estimate (also known as “magnitude” uncertainty), the activity level and emission factor had great uncertainties; however, we calculated the number of different emissions using the IPCC guidance, which had high confidence. Besides the magnitude uncertainty, the spatial weight distribution created and propogated the uncertainty. The uncertainty of *Weight*_*p*_ was the error in the generation of the population map. Uncertainty of *Weight*_*I*_ depended on identifying the accuracy of industry location and the “blooming” effect of night light images. The area of the road network couldn't represent the transport emission intensity [9].

Based on the research of Wang et al. [10], greater accuracy could be obtained by comparing the total estimated emissions with the total number of all grids on the map. The maps showed that the absolute errors of total emissions at R_30m_ and R_500m_ were 886.70 t and 0.28 t, respectively. The relative errors of total emissions at R_30m_ and R_500m_ were 0.005% and less than 0.001%, respectively, especially in residential emissions. RMSEs at R_30m_ and R_500m_ were 185.73 t and 4508.19 t, respectively.

### 2.3 Generation of urban form fragmentation

#### 2.3.1 Identification of urban functional districts

With reference to the research of Chi and Long [11], we generated urban functional districts using the land use map, master planning data and POI data that generated the grid frequency density (FD) and grid category ratio (CR).

FD and CR are defined as follows:

(7) $$F_{i}=\frac{n_{i}}{N_{i}}\left(i=1,2,3\cdots ,6\right)$$

(8) $$C_{i}=\frac{F_{i}}{\sum_{i=1}^{9}F_{i}}\times 100\%,i=1,2,3\cdots ,6$$

where, *F*_*i*_ represented the FD of the corresponding classification and *C*_*i*_ represented the ratio of the FD of the corresponding classification to the FD of all classifications in the grid; *i* represented the functional category of POI; *n*_*i*_ was the number of POI in the *i*th category in the grid; *N*_*i*_ represented the sum of POI in the *i*th category. If *C*_*i*_ exceeded 50%, we defined the grid as the single functional district patch, with the category being the same as the corresponding classification. If *C*_*i*_ was less than 50%, we defined the grid as the mixing functional district patch.

Since POI was point data, there were some data deficiencies in areas where human activity was rare. Moreover, there were only 10 categories of urban functional areas in the POI data, in which six functional area categories (Water, agriculture and forestry land, other non-construction land, village construction land, special land, mining land) were missing compared to the master plan map data. We spatially superimposed two kinds of data and found that the missing POI data area was labeled as “indefinite” and we replaced it using the master plan map [12]. The indefinite patch was corrected using the land use/cover data. The rules for correcting an indefinite classification of urban functional district patch were as follows:

To identify the parcels whose classification was inconsistent among the POI, land use map and master planning data. No correction was needed for parcels whose classification was all consistent, or at least classification of land use/cover and POI should be the same. The inconsistent parcels were defined as uncertain parcels and were then corrected. The uncertain parcels would be corrected by the land use/cover map. Due to the rare POI deficiency, the single functional district patches would be validated with the land use/cover map as well.

To confirm that urban functional districts were accurately defined, 100 verification points were randomly generated. The Google Earth image in 2013 was selected for visual identification. 84 verification points were consistent. Only 16 points were inconsistent. We revised the inconsistent urban functional district of these corresponding parcels into true urban functional subtypes. The urban mixed and single functional district/zone was defined according to the division of the mixture degree of each grid pixel.

#### 2.3.2 Landscape mixed index of urban functional districts

The equation used to calculate the urban functional landscape mixed index was as follows:

(9) $$Landscapemix_{i}=\frac{-\sum_{k=1}^{K}p_{k,l}ln\left(p_{k,l}\right)}{ln\left(K,l\right)}$$

where *K* was the number of urban functional districts types in the *l*th pixel and *p*_*k,l*_ represented the area percentage of the *k*-type urban functional districts in the *l*th pixel. The higher the urban functional districts mixed index was, the higher the fragmentation degree was. The 0 mixed indexes represented the single urban functional district, the area with other values was the mixed urban functional district. The urban functional distrcts landscape mixed indexes at two resolutions were showed in Fig. 2.

#### 2.3.3 Lacunarity index and feature scale determination

We used the Lacunarity index to quantify landscape heterogeneity and select feature scales [13]. The feature scales were the sizes of the moving windows used to calculate the landscape metrics. The landscape metrics refer to a simple quantitative indicator that enriches information on landscape pattern. The metrics reflect the landscape structural composition and provide information regarding some aspects of spatial configuration. Different moving window sizes were set for R_30m_ and R_500m_. As to R_30m_, the moving window size ranged from 30 m×30 m–9000 m× 9000 m. The moving window of R_500m_ was from 500-m×500-m to 10000 m ×10000 m. The Lacunarity index was calculated based on the following equation:

(10) $$\text{Λ}\left(r\right)=S_{s}^{2}\left(r\right)/\bar{S^{2}}\left(r\right)+1$$

where Λ(r) was the Lacunarity index; *S(r)* was the mean mixing degree contained in each sample with the moving window; *S*_*s*_^*2*^(*r*) was the variance and *r* was the length of the moving window. The inflection point of log-log curve of the size-Lacunarity index was the feature scale, i.e., the maximum value of the differential of the fitted hyperbolic function.

The logarithm of the Lacunarity index decreased as the logarithm of the size increased (Fig. 3). The logarithmic curve dropped greatly in the middle and right sections, indicating that the hierarchical structure of the whole urban mixed landscape and the fractal dimension distribution were in the middle and left sections. Polynomials were used to fit the porosity exponent double logarithmic curve at R_30__m_ and R_500__m_. The fitted hyperbolic curve showed that R^2^ reached 0.999, all the fitted equations were showed in Table 4, of which the fitting effect was excellent. The feature scales of functional area fragmentation analysis were 652.698 m (R_30__m_) and 1000 m (R_500__m_) (Fig. 4).

#### 2.3.4 Landscape metrics

To characterize the fragmentation degree of urban form, Fragstats 4.2 software was used to calculate the number of patches (NP), patch density (PD), division (DIVISION) and effective mesh size (MESH) metrics. Fragstats 4.2 was a spatial analysis program for categorical map and could calculate all the different landscape indexes at different levels. (https://www.umass.edu/landeco/research/fragstats/downloads/fragstats_downloads.html).

Formulas of the above mentioned indicators were as follows:

(11) $$NP=N_{i}$$

(12) $$PD=\frac{\sum_{i=1}^{M}N_{i}}{A}\left(\text{PD}>0\right)$$

(13) $$\text{ DIVISION}=\left[1-\sum_{j=1}^{n}{\left(\frac{a_{ij}}{A}\right)}^{2}\right]\left(0\leq DIVISION\leq 1\right)$$

(14) $$\text{MESH}=\frac{\sum_{j=1}^{n}{a_{ij}}^{2}}{A}\left(\frac{1}{10000}\right)$$

Where *N*_*i*_ was the number of patches in the *i*th landscape type (e.g. agriculture); *M* was the total number of landscape types; *A* was the total area of the landscape (m^2^); *n* represented the number of single patches (mixing degree was 0) in the landscape; *a*_*ij*_ was the area of patch *ij*.

High NP, PD and DIVISION values represented high fragmentation, whereas a high MESH value represented low fragmentation. We divided Jinjiang city into low-, mid- and high-fragmentation level areas according to classification maps obtained by the K-Means method (Fig. 5). K-Means method which is a quick clustering method has a low algorithm complexity and a high efficiency in handling the big data [14]. The range of different landscape metric values were showed in Table 5.

**Table 5:** The range of different landscape metric values at R_30m_ and R_500m_.

Spatial Resolutions	Metric Value	NP	PD	DIVISION	MESH
30 m	Low	4	10.078	0.117	2.094
	Medium	16	40.312	0.589	9.145
	High	24	60.469	0.770	16.319
500 m	Low	2	0.889	0.198	25.000
	Medium	4	2.667	0.568	58.333
	High	6	3.111	0.716	108.333

### 2.4 City-scale effect related impact factors of the CO_2_ mitigation: PUA and POID

The UFD was the relatively detailed function classification of built-up areas. Non-built-up areas can be treated as a single function classification. The UFD fragmentation of non-built-up areas was not significant enough. In contrast, the phenomenon of urban functional landscape fragmentation in urban built-up areas was more significant. In quantifying the influence of UFD fragmentation on urban CO_2_ emissions, we used PUA and POID to control the effects of the urban population, economic scale and aggregation on CO_2_ emissions, which are the three most recognized major impact factors of urban CO_2_ emissions.

PUA was expressed as the proportion of the urban area to the whole unit per grid. Detailed information on the metrics of urban sprawl was provided by Ref. [15]. POID was expressed as the number of POI to the whole units per grid. PUA was classified into three categories (high, low and middle) using K-Means. POID was classified into three categories (POID=0, POID=1 and POID >1).
