# Supplementary material for: A spatial database of CO2 emissions, urban form fragmentation and city-scale effect related impact factors for the low carbon urban system in Jinjiang city, China
Source: Data Brief. 2020 Feb 11;29:105274. doi: 10.1016/j.dib.2020.105274 (PMC7042417; doi:10.1016/j.dib.2020.105274)
Supplement: Multimedia component 1 [file mmc1.zip › 数据包/paper/Dai_et_al-SA2018-2018.pdf]

SPATIAL ACCURACY 2018

21-24 May 2018

Beijing, China

Proceedings of Spatial Accuracy 2018

Editors:

Yong Ge, Xin Li & Gerard Heuvelink

Organized by:

ISARA | International Spatial Accuracy Research Association

spatial-accuracy.org

High-resolution Mapping of Direct CO2 Emissions and Uncertainties at the Urban Scale

Shaoqing Dai [1,2], Shudi Zuo [1], Yin Ren*[1,3]

[1] Key Laboratory of Urban Environment and Health, Institute of Urban Environment, Chinese Academy
[2] University of Chinese Academy of Sciences, China
[3] Ningbo Urban Environment Observation and Research Station-NUEORS, Chinese Academy of Sciences, Ningbo, 315800, China

*Corresponding author: yren@iue.ac.cn

ABSTRACT

Mapping direct carbon emissions at high-resolution in urban environments could help in the development of measures to mitigate carbon emissions through optimizing the layout of inner structures. It requires the use of a mapping method combining the bottom-up and top-bottom calculations with uncertainty evaluations. This study developed a method for urban scale analyses of carbon emissions, including a theoretical framework of uncertainty distribution and transmission. Using Jinjiang City, China, as a case study, we applied this method to calculate the amount of carbon emissions in grids distributed across a city. This information was used to analyze emission uncertainties and its sources. The calculated emissions were allocated through the accurate spatial identification of three emission sectors and proxy data. Two different population spatialization methods were constructed in order to create 30 m and 500 m resolution grid maps. We designed four different Monte-Carlo simulation scenarios to analyze the uncertainties of the two maps. The results showed that the method developed here was suitable for delineating carbon emissions at the urban-scale. The 30 m resolution map showed that residential emissions were widely distributed, whereas industrial emissions were more concentrated, with the opposite trend being detected in the 500 m resolution map. Calculations of carbon inventory and spatial proxy had more impacts on the 30 m resolution map than on the 500 m resolution map. During the process of spatial superposition, the uncertainties from different sectors showed a nonlinear relationship, which was represented by smaller total uncertainties compared with the sum of uncertainties from the three emission sectors. In conclusion, this study provides important baseline data that could be used to optimize urban form by promoting low-carbon city construction.

KEYWORDS: Monte-Carlo simulation; spatial proxy; carbon tabular inventory; uncertaint y propagation; bottom-up and top-bottom

I. TABLES AND FIGURES

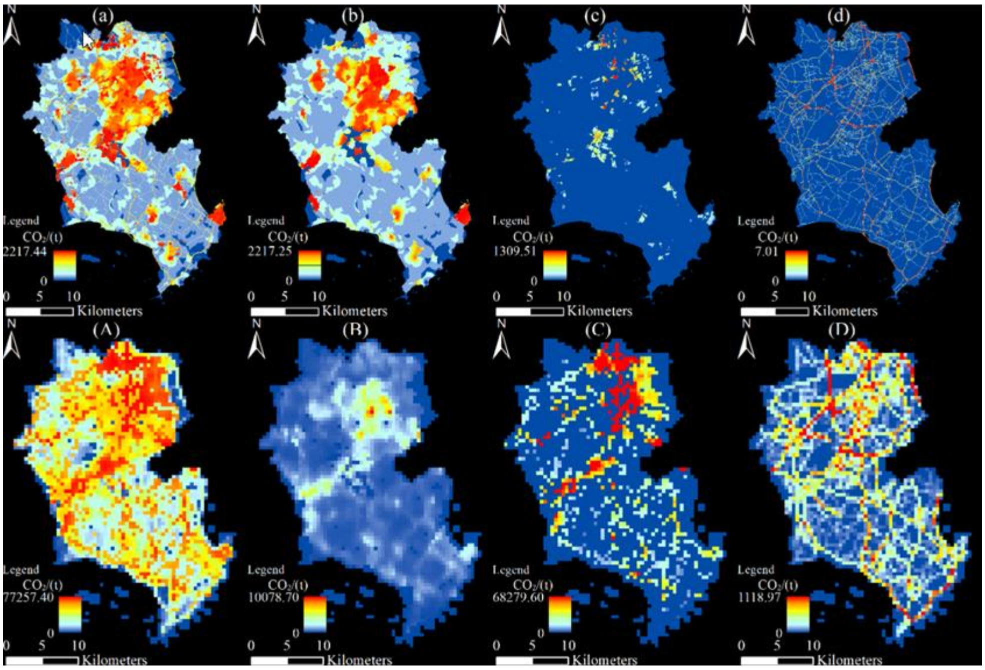

Figure 1: Map showing CO2 emissions at 30 m and 500 m resolution. The upper figures are at 30 m resolution, the lower figures are at 500 m resolution. (a) and (A) are the total direct CO2 emissions maps, (b) and (B) are the residential CO2 emissions maps, (c) and (C) are the industrial CO2 emissions maps, and (d) and (D) are the traffic CO2 emissions maps.

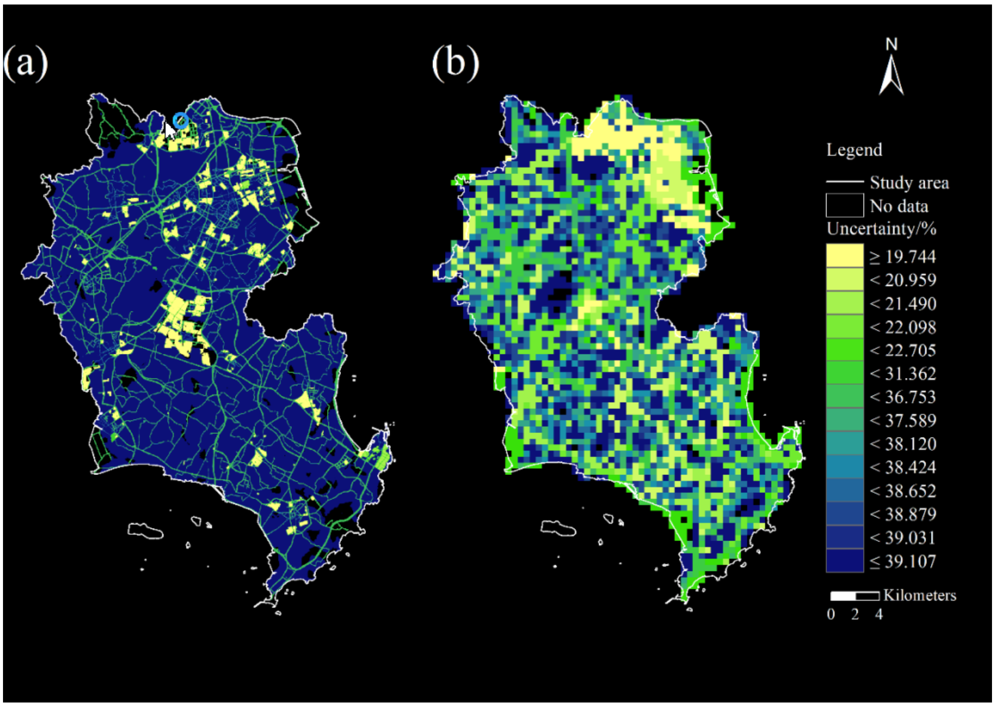

Figure 2: Map showing the uncertainty in activity levels. (a) and (b) represent the uncertainty maps of total CO2 emissions at 30 m and 500 m resolution, respectively.

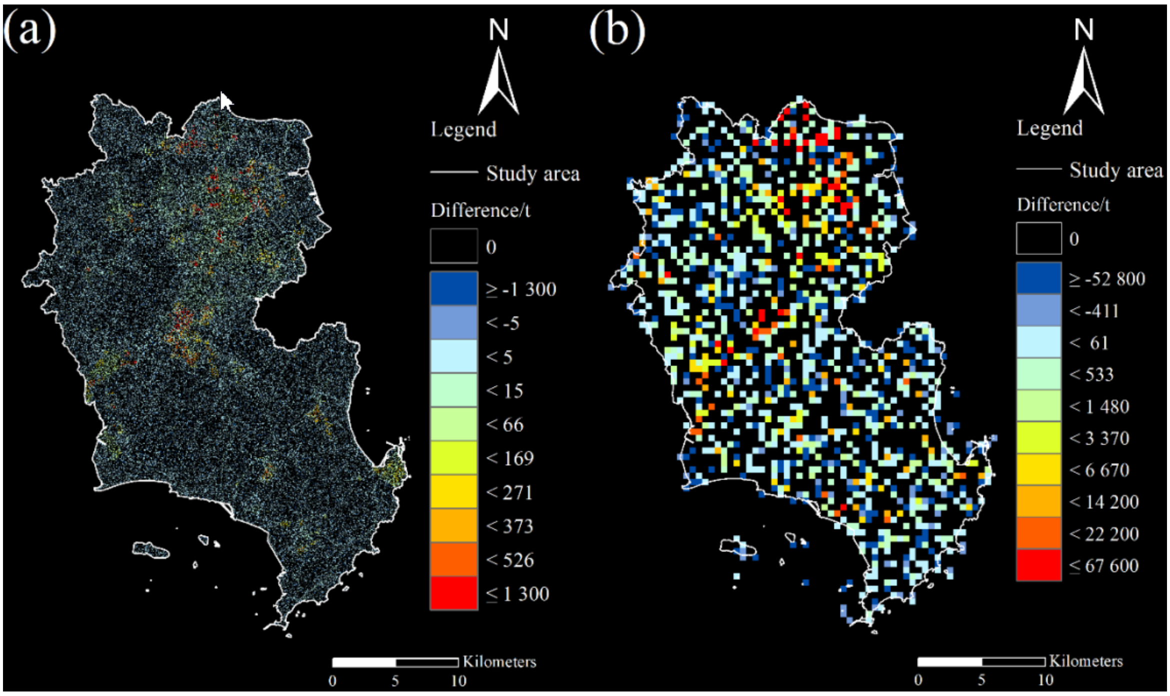

Figure 3: Map of uncertainty of spatial proxy, (a), (b) represent the uncertainty map of total CO2 emission at 30 m and 500 m

II. FORMULA

In terms of the IPCC accounting methodology, we calculated direct carbon emissions (Sco pe 1), from which we created two high-resolution CO2 emission maps of different scales (30 m and 500 m) by combining spatial models with top-bottom and bottom-up approache s. Based on Monte Carlo simulations, four different schemes were designed to study the uncertainty, including the overall uncertainty of carbon emissions from different sectors (sc heme 1 and 2) and how uncertainties influence different procedures of grid mapping and the superimposed delivery process (scheme 3 and 4).

$$\boldsymbol{Grid_{i,CO_2} = f(c_l, weight_{i,l})} \tag{1}$$

Where $Grid_i$, CO_2 is the CO_2 emission value on the i th grid (i = 1,2,3 ..., n), C_l re present the total amount of CO_2 emissions from the different CO_2 emission sources (l = r es, ind, trans), $Weight_{i,l}$ is the weight of the l type of emissions on grid i.

$$\boldsymbol{Uncertainty = \frac{CI_{95}}{\overline{CO_{2,N}}}, CI_{95} = \frac{CO_{2,97.5} - CO_{2,2.5}}{2}} \tag{2}$$

where CO2,97.5 is the 97.5% quantile of CO2 emissions in the Monte-Carlo simulation, CO2,2.5 is the 2.5% quantile of CO2 emissions in the Monte-Carlo simulation, and $\overline{\boldsymbol{CO_{2,N}}}$ is the mean value of direct CO2 emissions from the Monte Carlo Simulation.

$$\textbf{error} = \frac{\sum_{i=1}^{n} \boldsymbol{Difference_{i,1}}}{\boldsymbol{C_l}} = \frac{\sum_{i=1}^{n} |\boldsymbol{realvalue_{i,l} - simulation_{i,l}}|}{\boldsymbol{C_l}} \times \mathbf{100\%} \tag{3}$$

where $Difference_{i,l}$ is the absolute value of the difference between the real pixel and simu lation value emission from *l* th sector on the *i* th grid (l = 1,2,3,4, which represents the residential, industrial, transport and total emissions). Cl is total CO2 emissions from *l* th s ector.
