# Supplementary material for: A spatial database of CO2 emissions, urban form fragmentation and city-scale effect related impact factors for the low carbon urban system in Jinjiang city, China
Source: Data Brief. 2020 Feb 11;29:105274. doi: 10.1016/j.dib.2020.105274 (PMC7042417; doi:10.1016/j.dib.2020.105274)
Supplement: Multimedia component 1 [file mmc1.zip › 数据包/paper/Zuo_et_al-JCLP-2019.pdf]

Journal of Cleaner Production xxx (xxxx) xxx

Contents lists available at ScienceDirect

Journal of Cleaner Production

journal homepage: www.elsevier.com/locate/jclepro

More fragmentized urban form more CO_2 emissions? A comprehensive relationship from the combination analysis across different scales

Shudi Zuo [a, b, *], Shaoqing Dai [a, b], Yin Ren [a, c, **]

[a] Key Laboratory of Urban Environment and Health, Institute of Urban Environment, Chinese Academy of Sciences, Xiamen, 361021, China
[b] University of Chinese Academy of Sciences, Xiamen, 361021, China
[c] Ningbo Urban Environment Observation and Research Station-NUEORS, Chinese Academy of Sciences, Ningbo, 315800, China

A R T I C L E I N F O

Article history:
Received 26 April 2019
Received in revised form
27 September 2019
Accepted 29 September 2019
Available online xxx

Handling Editor: Yutao Wang

Keywords:
Urban functional landscape
Aggregation effect
Fossil fuel CO_2 emission
Spatial analysis
Spatial resolution

A B S T R A C T

Scientifically delineating the spatial heterogeneity of urban landscape fragmentation in relation to CO_2 emissions helps the urban carbon mitigation strategy. The combination analysis across spatial resolutions, which is rare, helps explore the comprehensive relationship between urban fragmentation and CO_2 emissions. This study compared the relationships between urban form fragmentation and CO_2 emissions in an urban system through the analytic framework composed of the Pearson correlation analysis, geographically weighted regression (GWR), and geographical detector methods with the use of multi-source data to construct the CO_2 emissions maps. As the result, there was less fragmentation with a 500-m spatial resolution (R_{500m}) than with a 30-m spatial resolution (R_{30m}). In terms of the GWR analysis, the coarse resolution resulted in: 1) positive coefficients of fragmentation metric becoming negative, and 2) greater absolute values of negative coefficients. As to the results of Geographical detector, single factor impact powers and interactions among fragmentation factors showed a weakening effect at R_{30m}, but a strengthening and weakening effect at R_{500m}. However, there were common results observed in low-fragmented areas across different scales. That is, in low-fragmented mixed-function areas and industrial areas, the more fragmented the area was, the less the CO_2 emission there would be. However, in low-fragmented residential, administrative and public service areas, the more fragmented the area was, the higher the CO_2 emission there would be. Therefore, the government should disperse the mixed function zones and industrial parcels with diverse types of land, and build the contiguous residential and public service land in the low fragmentation area of urban system. The results of this study can provide a reference for the other small and medium towns and cities. The analytical framework can be applied to CO_2 emissions research in urban agglomerations, megacities, and small towns.

© 2019 Elsevier Ltd. All rights reserved.

1. Introduction

Besides the industrial production, transportation, local weather, and fossil fuel use (Wang et al., 2012, 2016b, 2016c; Wang et al., 2018b), urban form is the factor that affect energy-related CO_2 emissions from human activities secondarily (Wang et al., 2014, 2017). With urbanization, the urban economic scale effect becomes a major driving force for intensive human activity and then the emission. Except for that, about 50% of urban CO_2 emissions have been attributed to urban forms, mixed land use, building types, and transportation networks (Christen et al., 2011). The major forms of human activity affected by urban form are traffic and residential energy consumption/use emissions (Lee and Lee, 2014). With respect to curtailing urban CO_2 emissions, scholars not only recommend to improve energy efficiency and reduce energy emissions per unit of gross domestic product (GDP). Inquisitorial discussions have led scholars to shift their focus more toward the favoring of urban form features that can change human behavior to produce less CO_2 emissions, such as through public policy

* Corresponding author. Key Laboratory of Urban Environment and Health, Institute of Urban Environment, Chinese Academy of Sciences, Xiamen, 361021, China.
** Corresponding author. Key Laboratory of Urban Environment and Health, Institute of Urban Environment, Chinese Academy of Sciences, Xiamen, 361021, China.
E-mail addresses: sdzuo@iue.ac.cn (S. Zuo), yren@iue.ac.cn (Y. Ren).

https://doi.org/10.1016/j.jclepro.2019.118659
0959-6526/© 2019 Elsevier Ltd. All rights reserved.

initiatives for the development of compact cities, improvement of public transportation (Creutzig et al., 2015). After a wide examination of the relationship between urban higher fragmentation and CO_2 emissions, scholars found fragmentation of land use areas (i.e. areas designated for human activities or purposes) is associated with greater CO_2 emissions (Girvetz et al., 2008; Gong et al., 2013). However, the evidences might be biased by the uncertainties from the spatial distribution of emissions (also referred to "downscaling", or "representation" uncertainty), the delineation of urban fragmentation landscape, and the analysis method which ignores the spatial heterogeneity.

Urban form fragmentation, one of the landscape metrics to assess the spatial configurations and arrangements of human activities characteristic of an urban region, is dependent upon local geographical features and the spatial distribution of functional areas. Urban form fragmentation reflecting the interference level of human activity has a bidirectional interaction with environmental factors, being both shaped by the environment while also having direct and indirect anthropomorphic impacts upon the environment (Fang et al., 2015; Liu and Sweeney, 2012; Nordbo et al., 2012; Owens, 1986). Previous studies using panel data from the statistic yearbook only analyzed direct linear relationships of CO_2 emissions with fragmentation. For instance, Ou et al. found that the fragmentation of built-up areas and urban sprawl was associated with higher CO_2 emission levels (Ou et al., 2013). Such findings have implications for emission reduction policies at the national and provincial level. However, linear analyses of the relationship between urban form indicators and CO_2 emissions is insufficient (Ren et al., 2016).

Moreover, the impacts of scale effect (e.g. spatial resolution) would result in the uncertainty of the conclusion. There were two aspects affected by the spatial resolution problem. The first one was the spatialization process of CO_2 emission grid map. The downscaling or representation uncertainties exist because of the mismatching of the spatial scales for the real emission source activities and the generated emission boundary or grid. Since the real emissions would be downscaled to a grid format using spatial proxies which are believed to be representative. The grid size (spatial resolution) might "smooth out" the point emission intensity. Gately and Hutyra found that the residential and total CO_2 emissions were insensitive to this resolution variation, but the road and power plant CO_2 emissions were opposite for the spatial resolutions larger than $1\,km \times 1\,km$ (Gately and Hutyra, 2017). Furthermore, there were relatively high uncertainties of the regional fossil fuel carbon dioxide inventories (mean percent relative difference of 33%–78% for five popular inventories) at urban scale (Gately and Hutyra, 2017) and few detailed statistical yearbooks of CO_2 emission inventory data for urban scale in China. Nevertheless, employing a 1 km grid map to examine CO_2 emissions in the urban system of Tianjin metropolis, Cai et al. applied various defined urban borders and found differences in the spatial heterogeneity of CO_2 emissions within the urban area, which led them to propose more detailed and effective emissions reduction policies (Cai and Zhang, 2014).

The second one was the description level of the urban form fragmentation. So far, the conclusion that more fragmented urban landscapes produce greater CO_2 emissions has been derived mostly from studies employing cursory land use classification with a spatial resolution of 1 km or worse (Xia et al., 2017). The lack of detailed information related to functional districts within urban areas can bias analytic results. Being an ecosystem that benefits human beings, the urban landscape consists not only natural elements, but also of artificial elements. Urban land can be used for commerce, industry, residence, transportation, and public land uses, each of which contributes to the urban functional landscape (Lin et al., 2016). The grid map with 1 km or larger spatial resolution is not detailed enough to reflect the pronounced spatial heterogeneity of urban areas. Because small patches are "lost" or merge with bigger ones as pixel grain size increases, resulting in the mean size of the remaining patches greatly increasing. Scholars usually used the popular landscape fragmentation metrics to characterize landscape fragmentation patterns. To be noticed that, some of them are very sensitive to the spatial resolution variation, such as number of patches (NP) and patch density (PD). The insensitive metrics described the area information of certain landscape type, such as division (DIVISION) and effective mesh size (MESH). In this study, we chose the stable index MESH to analyze the relationship. However, it is not yet known whether analyses of higher resolution data would lead to the same conclusion (Girvetz et al., 2008).

Previous studies have enabled the closer study of urban landscape patterns and environmental effects, though the existence of correlations between morphology/land fragmentation and CO_2 emissions does not ensure that modifying morphology parameters or fragmentation levels will reduce CO_2 emissions because it is likely that the correlation is non-causal. Moreover, it is possible that urban planning guided by this conclusion may have negative effects. Small green belts and water patches within urban areas, which can introduce fragmentation with respect to landscape indicators, can provide important ecological functions, such as cooling, noise reduction, air purification, as well as cultural functions, such as entertainment sites (Gong et al., 2013). Even in a highly fragmented region, green land and water space accessibility can reduce the CO_2 emissions related to residential energy consumption (Ye et al., 2015). Thus, the functionality of spaces within urban landscapes and the actual conditions within and around such spaces should be considered in urban planning decisions. That is, the relationship between CO_2 emissions and fragmentation of functional landscapes should be assessed after accounting for the effects of economic aggregation and urban sprawl. Therefore, the interaction among various factors affecting the spatial distribution of urban CO_2 emission could lead to the analysis bias (Wang et al., 2016a). Finally, multiple economic and urban characteristic factors can impact CO_2 emissions significantly, including GDP, population density, resident income levels, and land use intensity (Creutzig et al., 2015; He et al., 2017; Wang and Liu, 2017; Wang et al., 2016d; Zhang et al., 2017).

Facing the above mentioned uncertainties of three aspects and the shortcoming of the unsuitable applied at the city level, this study proposes the multi-scale analysis framework (30-m and 500-m) to comprehensively quantify the relationship between the CO_2 emission, urban fragmentation and urban economic aggregation effect with the aim of gaining information that will be useful for policies guiding low-carbon city construction. The framework considers surface heterogeneity in explorations of spatial effects on explanatory variables and also assesses interactions among explanatory variables and conducts quantitative analyses of categorical variables, which provides an effective means of clarifying the mechanism underlying urban spatial morphological changes (Wang et al., 2010). Furthermore, we propose two metrics for human activities instead of the population density and GDP spatial distribution to represent the urban aggregation effect. Because GDP spatial distribution and population density are usually mapped by the statistics data and the interpolation method, they are not the real observation data. While the density of points of interest (POID), a new indicator of human activities, has gained attention as a characteristic of high spatiotemporal resolution (Cai et al., 2017). Except for this indicator, the proportion of urban built-up area (PUA) used to characterize the urban land use intensity represents the human activities as well. Finally, based on the functional district pattern of the central area of an urban region (i.e., the urban system

Please cite this article as: Zuo, S et al., More fragmentized urban form more CO_2 emissions? A comprehensive relationship from the combination analysis across different scales, Journal of Cleaner Production, https://doi.org/10.1016/j.jclepro.2019.118659

of Jinjiang city, China), the conclusion on the common results across different scales was drawn.

This study intends to: 1) propose the linear and nonlinear analysis framework for understanding the relationship between CO_2 emissions and urban fragmentation by considering the potential impact factor — spatial effects including spatial autocorrelation and heterogeneity, which can be determined by spatial regression modeling (e.g. geographically weighted regression, GWR) and geographical detector model; 2) provide the urban form design guidance for the city mangers by exploring the impact of 30-m versus 500-m spatial resolution maps. The present study allows us to overcome the uncertainties sources from the CO_2 emission grid mapping, urban functional fragmentation delineation and spatial heterogeneity through the multi-scale geostatistical analysis framework.

2. Case study area

Jinjiang is located in southeastern China, and covers an area of 649 km^2, with 121 km lining the coast (118° 24′E–118° 43′E, 24° 30′N-24° 54′N). Jinjiang ranked the 5th position comparing with other 100 developed counties, making it representative of the local urbanization model, which is accompanied by fast economic growth (total GDP: 136.39 billion RMB in 2013) and urban sprawl, as well as a stable permanent population (1.09 million in 2013) and a large floating population (1.24 million in 2013). From the early 1990s, its industrial output exceeded 50% of the total GDP. From 2015 to 2017, the economic growth rate remained at about 7.8%–8.6%, the total GDP reached 162.04 to 198.15 billion RMB.

3. Methodological framework

We proposed a methodological framework used for determining the relationship between impact factors and geographical/ecological variable. In this case study, the variable was CO_2 emissions and impact factors included the pattern of fragmentation, PUA and POID. Our framework consists of three progressive components: (1) Pearson correlation analysis was used to describe the linear relationship between the pattern of fragmentation, PUA, POID versus the pattern of CO_2 emissions. (2) GWR was used to explore the linear strength and direction of influence of the fragmentation indicator, PUA, POID. This model identified different directions and regions where fragmentation, PUA, POID impacted CO_2 emissions. (3) Geographical detector model was used to quantify the nonlinear impact powers of fragmentation, PUA, POID and the interaction powers of all the factors. The hierarchical results of the three methods were used to understand how the tendency and location of increasing urban landscape fragmentation affected urban CO_2 emissions, and included the interactions of fragmentation and other CO_2 emissions impact factors (Fig. 1).

GWR targeted for the linear spatial analysis, and geographical detector model designed for the nonlinear spatial analysis are complementary. GWR is suitable for exploring the problem about spatial autocorrelation and temporal-spatial scales (Brunsdon et al., 1998). It enables the methodological framework to integrate spatial statistical analysis method based on locality. Moreover, the spatial variant coefficients of the whole area obtained from GWR help make inferences about spatial process. As one of the most effective methods to solve the problem of spatial heterogeneity, the geographical detector model not only explores the spatial influence of explanatory variables on explained variables, but also understands the interaction between each explanatory variable. Moreover, it's particularly good at analyzing type variables and requires the categorical variables as the input data. (Wang et al., 2010).

Y represents the phenomenon (numeric independent), X_i and $X_i^{'}$ represents the environment factors (numeric dependent and category dependent respectively), $r_{x,y}$ represents the correlation coefficient. We collected all the data like the middle section in Fig. 1 and used three methods (step 1: Pearson correlation analysis, step 2: Geographical weighted regression, step 3: Geographical detector) to quantify the relationship between the phenomenon and environment factors.

3.1. Research data

Research data in this study included the spatial distribution map of CO_2 emissions, pattern of fragmentation, PUA and POID. Here we used a multi-sources data fusion approach and a classical landscape ecology analysis method described details in the data in brief paper (Dai et al., 2019). The multi-sources data included the vector master planning spatial data for Jinjiang 2010–2030, Baidu website point of interest (POI) data, road network data from 2013, visible infrared imaging radiometer data from the joint NASA/NOAA Suomi National Polar-orbiting Partnership, total industry GDP (ten thousand yuan), energy consumption per industrial output value (standard coal/ten thousand yuan), the Jinjiang National Economic and Social Development Bulletin, the Transportation Development Statistics Bulletin and vector population spatial data from 2013.

We used a combined global downscaled and bottom up approach to estimate the gridded CO_2 emissions. Furthermore, POI, road network data and master planning spatial data were used for identification of urban functional land/zone, PUA and POID. See the data in brief paper (Dai et al., 2019) for the details, please.

3.2. Model theory

The detailed information of model which was used in the methodological framework was introduced in this section.

The GWR model is widely used in spatial econometrics, criminal exploration, environmental pollution mapping, environmental health, and NPP estimation (Bitter et al., 2007; Cahill and Mulligan, 2007; Hu et al., 2012, 2013; Wang et al., 2005). It expanded the linear regression model, so that the regression coefficient β changed with the spatial position of i, reflecting how the explanatory variables impact the explanatory variables that changed with spatial position, as:

$$y_i = a_0(u_i, v_i) + \sum_k a_k(u_i, v_i)x_{ik} + \varepsilon_i \quad \text{(Equation 1)}$$

where y_i is the dependent variable at point i, x_{ik} is the value of the k-th independent variable at point i, ξ_i is the residual, (u_i, v_i) is the spatial coordinate of the i-th sample point, and $a_k(u_i, v_i)$ is the value of the continuous function a_k (u_i, v_i) at point i. The continuous function a_k is a function of distance attenuation, where the Gaussian kernel function is used and the bandwidth is the feature scale. Considering the strong collinearity among the landscape metrics, the MESH, which is not sensitive to the scale, is chosen as the independent variable (Gao and Li, 2011).

Geographical detector (http://www.geodetector.org/) is initially used to analyze local disease risk and related geographical factors (Wang et al., 2010). The Factor Detector module is based on spatial heterogeneity, whereby it is assumed that geographical objects always exist in specific spatial locations, and the environmental factors that affect their development are spatially different. If changes in the environmental factors and geographical objects are consistent in space, the factors significantly affect the occurrence and development of geographical objects, in which:

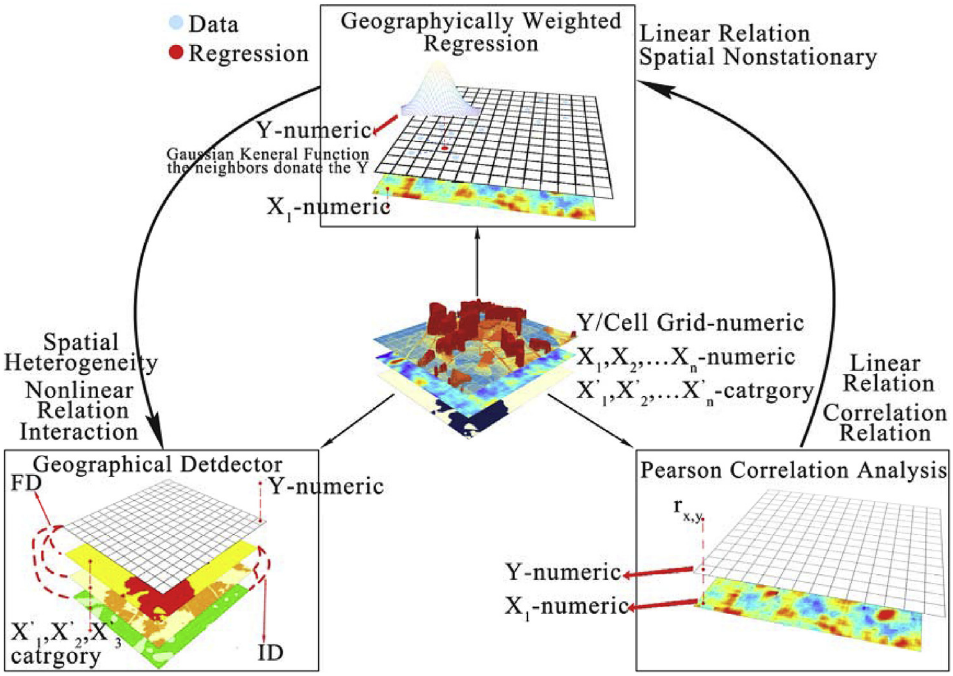

Fig. 1. Methodological framework.
Abbreviations: FD, Factor Detector; ID, Interaction Detector.

$$q = 1 - \frac{\sum_{h=1}^{L} N_h \sigma_h^2}{N\sigma^2} \quad \text{(Equation 2)}$$

where q is the impact power; $h = 1$; L is the stratification of factor X (here, it is the degree of fragmentation, PUA, POID); N_h and N are the number of units in layer h and whole study region, respectively; $\sigma^2{}_h$ and σ^2 are the variances of dependent variable values of layer h and the whole region. Unlike traditional statistical analysis, geographical detector measure interaction impacts based on the impact power of the new factor layer created from the spatial overlay of the two existing factors. By comparing the new impact power with the two-single effects, the principle of the interactive detector module is:

$$\left.\begin{array}{lll} \text{Weaken, nolinear}-: & & q(X_1 \cap X_2) < \text{Min}(q(X_1), q(X_2)) \\ \textit{Weaken, uni}-: & \text{Min}(q(X_1), q(X_2)) & < q(X_1 \cap X_2) < \text{Max}(q(X_1), q(X_2)) \\ \textit{Enhance, bi}-: & & q(X_1 \cap X_2) > \text{Max}(q(X_1), q(X_2)) \\ \textit{Indepent}-: & & q(X_1 \cap X_2) = q(X_1) + q(X_2) \\ \textit{Enhance, nonlinear}-: & & q(X_1 \cap X_2) > q(X_1) + q(X_2) \end{array}\right\} \quad \text{(Equation 3)}$$

where X_1 and X_2 are different existing factors. The total CO_2 emission is the dependent variable. The fragmentation of the functional district, PUA and POID represent independent variables. First, the fragmentation landscape metrics in each grid were classified by the K-Means clustering method. K-Means method which is a quick clustering method has a low algorithm complexity and a high efficiency in handling the big data (Hartigan and Wong, 1979). Next, the distribution layers of all the impact factors and CO_2 emissions were then loaded in ArcGIS. Finally, we combined the attributes of the CO_2 emission layer and all of the impact factors into one layer, and put all the data into the model and ran it. We also incorporated POID and urban expansion factors (PUA) (see section 2.3.5 of the data in brief (Dai et al., 2019) for details) into the Geographical detector model.

4. Results

4.1. High resolution spatial pattern of CO_2 emissions

The total amount of CO_2 emissions of residents, industry, and traffic emissions was 25.96 billion t (15.62% of total emissions), 137.29 billion t (82.59%), and 2.98 billion t (1.79%), respectively. With the total emission of 166.23 billion t, per capita annual CO_2 emissions were 8.12t/person, and the unit GDP CO_2 emissions were 1.219 t/10 thousand yuan RMB. The spatial accuracy of the population was relatively high at both 30-m and 500-m, and the fitting equation R^2 of the simulated and true values exceeded 0.98. The absolute and relative errors of CO_2 emissions at the 30-m scale were 886.70 t and 0.005%, respectively. The absolute and relative errors of CO_2 emissions at the 500-m scale were 0.28 t and <0.001%, respectively. The root-mean-square error (RMSE) of residential CO_2 emissions was 185.73 t at 30-m, while the RMSE was 4508.19 t at 500-m. CO_2 emissions in the city were high and spatially aggregated at both scales, with high fragmentation primarily occurring in the northeastern and middle parts. Low-emission areas were

ARTICLE IN PRESS

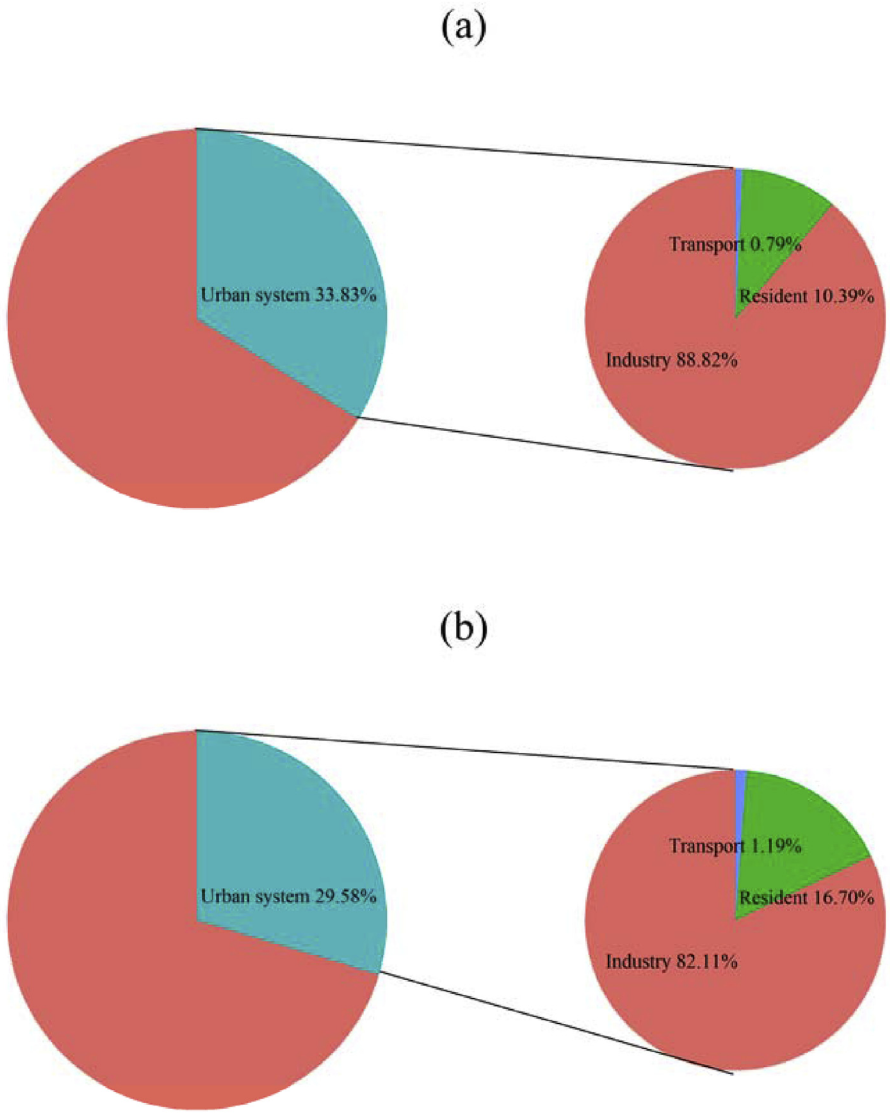

Fig. 2. Percentages of CO_2 emissions in Jinjiang attributed to total built-up areas and their composite functional land uses at R_{30m} (a) and R_{500m} (b).

mainly concentrated in the central and northern mountains and coastal areas (Fig. 3).

The boundaries of urban built-up areas (Fig. 3) were extracted from the urban land area of land use data. The built-up areas and CO_2 emissions differed slightly between the 30-m resolution (R_{30m}) and 500-m resolution (R_{500m}) scale data due to a spatial aggregation effect, with 7.10% and 11.12% of the total Jinjiang area being classified as built-up in the R_{30m} and R_{500m} assessments, respectively. Although built-up areas accounted for roughly a tenth of the total area, they were the source of 30% of the total CO_2 emissions. The proportions of carbon source origins within the built-up areas resembled the proportions for the entire city, with >80% of emissions being of industrial origin, some 10–20% being of residential origin, and <2% being of road traffic origin (Fig. 2).

4.2. Urban landscape fragmentation and city aggregation effects

The urban system, which consisted of various functional districts (Fig. 4) and had substantial fragmentation of its functional districts was confirmed to produce most of the city's CO_2 emissions. Therefore, we conducted further analysis of factors in the urban system.

As reported in Table 1, NP, PD, and DIVISION values in the urban system were greater for the R_{30m} dataset than for the R_{500m} dataset, while the MESH value was less for the former than the latter. Thus, greater fragmentation was observed at R_{30m} than at R_{500m}. The spatial distribution of landscape indexes also varied according to resolutions (Fig. 5).

At R_{30m}, the spatial distributions of NP and PD were similar, while the spatial distributions of DIVISION and MESH were opposite, as would be expected given that the latter two indicators are inversely correlated. In Fig. 5(a), we observed numerous patches with low-level fragmentation, especially in the urban center, and these patches showed a continuous distribution. There were some patches of low-level fragmentation dispersed in the periphery of study area. The patches of mid- and high-level fragmentation were interspersed with patches of low-level fragmentation. In Fig. 5(c), there were few, widely dispersed patches of low-level fragmentation. However, there were numerous patches of high-level fragmentation and distributed throughout the urban system. The patches of mid-level fragmentation were found surrounding those of low-level fragmentation.

At R_{500m}, the spatial distributions of NP and PD were distinct from each other, whereas the spatial distributions of DIVISION and

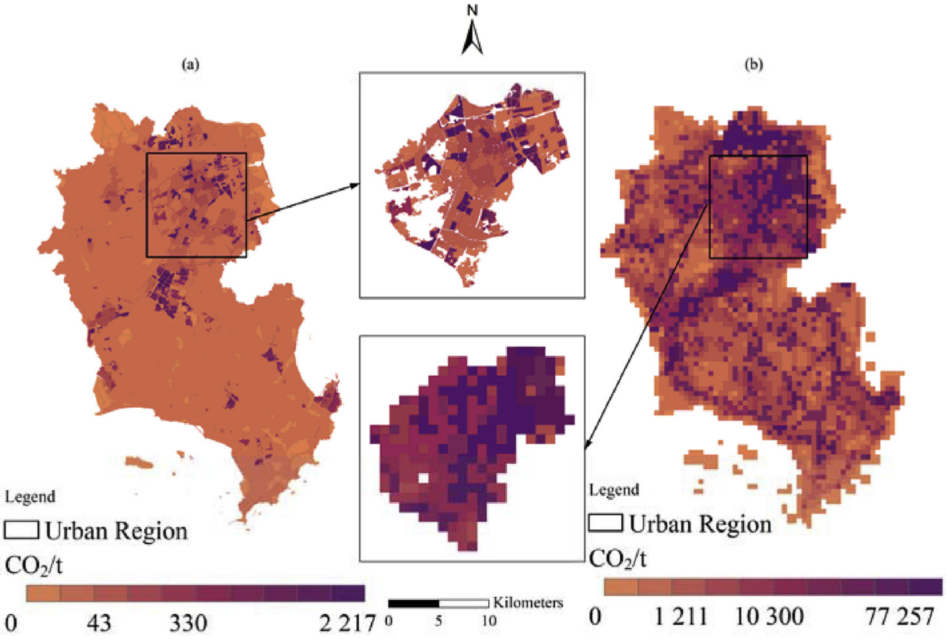

Fig. 3. CO_2 emissions distribution maps at R_{30m} (a) and R_{500m} (b).

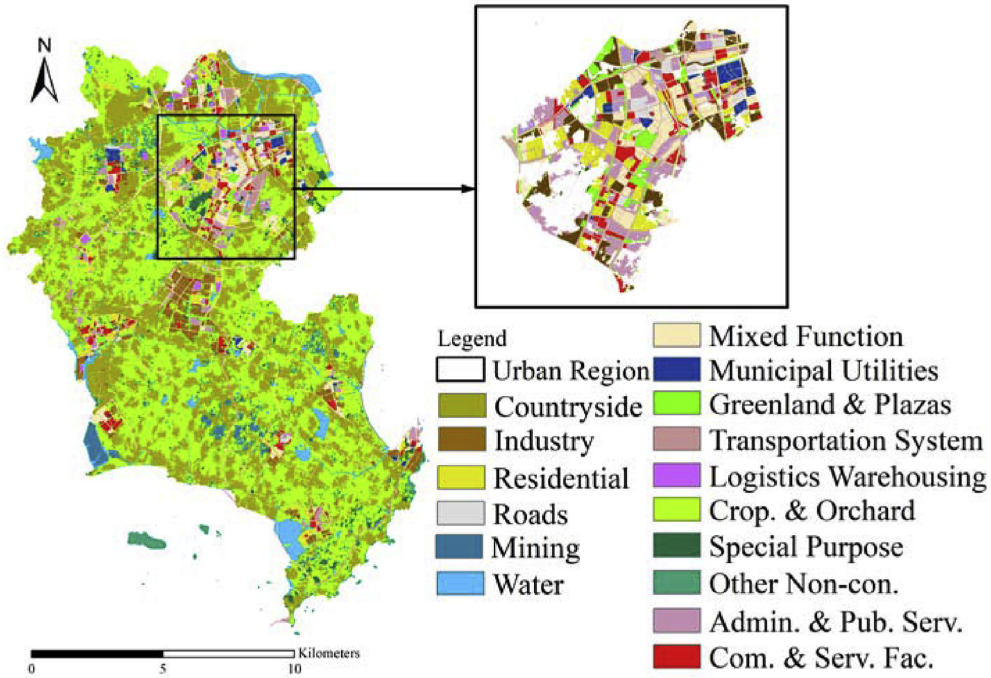

Fig. 4. Spatial distribution of urban functional districts (UFDs).
Abbreviations: Corp. & Orchard, Cropland and Orchard; Other Non-con.; Other Non-construction, Admin. & Pub. Serv., Administration and Public Services; Com. & Serv. Fac., Commercial and Service Facilities.

Table 1
Mean fragmentation metric values of functional district patches.

Metric	Resolution	
	30 m	500 m
NP	20.56	4.94
PD	51.79	2.20
DIVISION	0.76	0.69
MESH	9.48	69.20

MESH remained opposite. In Fig. 5(e), we observed a large number of the patches of mid- and high-level fragmentation. The patches of low-level fragmentation were dispersed, and a large patches of low-level fragmentation was found to be located in the central area. In Fig. 5(f), the number of patches of low-level fragmentation was large and the patches emerged with a broad distribution. The patches of mid- and high-level fragmentation showed a banded distribution. In Fig. 5(g), there were numerous patches of high-level

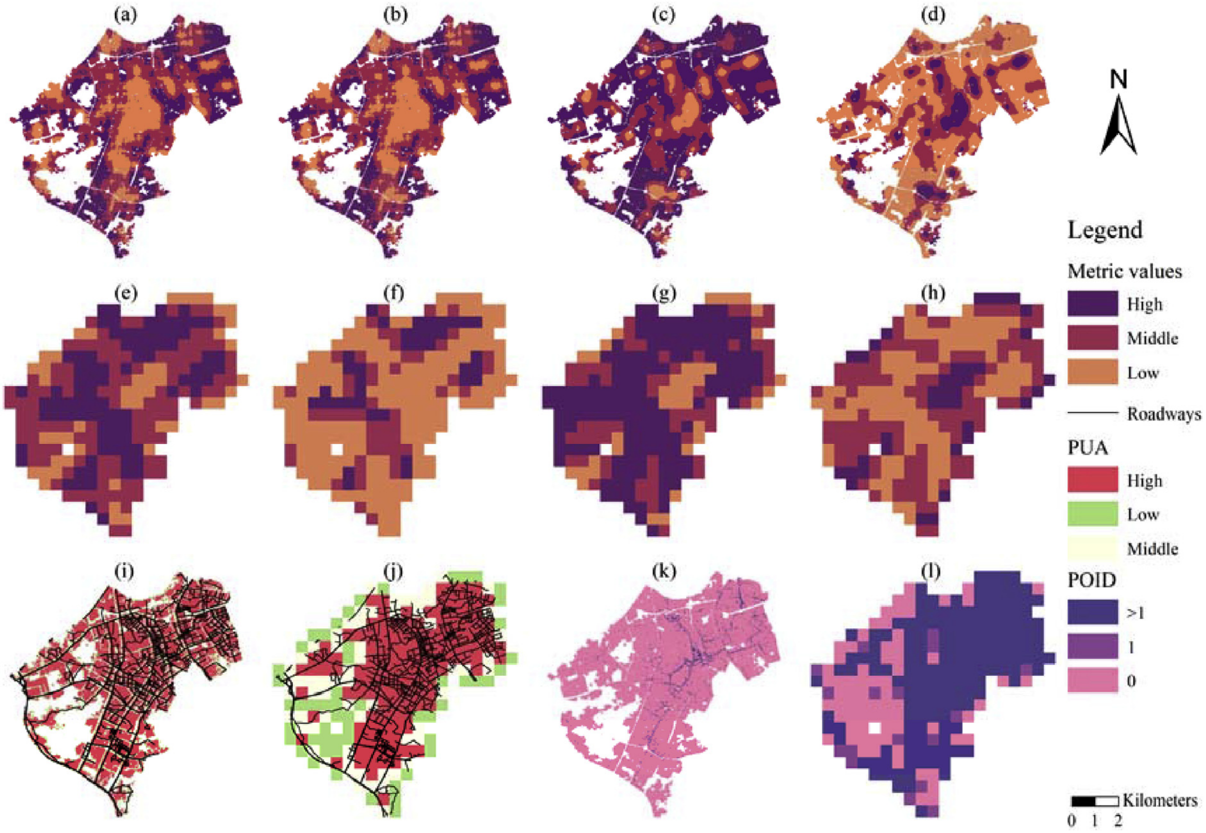

Fig. 5. Fragmentation, PUA and POID levels based on landscape metrics. The top row shows NP (a), PD (b), DIVISION (c) and MESH (d) fragmentation at R_{30m}. The middle row shows NP (e), PD (f), DIVISION (g) and MESH (h) fragmentation at R_{500m}. The bottom row shows PUA (i and j) and POID (k and l) at R_{30m} and R_{500m} (respectively). The ranges of different landscape metric values of different levels at R_{30m} and R_{500m} can be found in the table 4 of the data in brief (Dai et al., 2019).

fragmentation, but relatively few of low- and mid-level fragmentation. Most of the patches were dispersed, but a large patches of low-level fragmentation (Fig. 5(g)) coincided with the large centrally located patches (Fig. 5(e)).

The urban aggregation effects were evident in PUA and POID spatial distributions. At R_{30m}, the spatial patterns of PUA and POID followed roadways, with high-level POID areas concentrated on both sides of roadways, while PUA areas were distributed along roads. At R_{500m}, high-level POID and PUA areas accounted for most of the urban system with similar distributions.

4.3. *Statistical relationship of urban functional landscape fragmentation with CO_2 emissions*

4.3.1. *Classical statistical regression analysis*

Pearson correlation analysis (Appendix A Table S1) revealed weak correlations of UFD fragmentation with total CO_2 emissions and CO_2 emissions from particular sources (coefficients < 0.3) at R_{30m}. Fragmentation of UFDs had positive effects on the total, industrial, and, especially, residential CO_2 emissions. The pattern of results at R_{500m} was inverted relative to those at R_{30m}, with fragmentation of UFDs having a negative effect on CO_2 emissions.

With respect to the impacts of urban aggregation effects, at R_{30m}, PUA correlated weakly with total, residential and industrial CO_2 emissions, but correlated strongly with transportation CO_2 emissions (coefficient, −0.663). At R_{30m}, POID did not have any significant correlations with CO_2 emissions. At R_{500m}, both PUA and POID correlated weakly with all CO_2 emission measures.

4.3.2. *Geographically weighted regression*

As reported in Table 3, at R_{30m}, Akaike information criterion (AIC) values were lower for the GWR results than for the simple linear regression model (SLM). At R_{500m}, AIC values were slightly lower for the GWR model than for SLM, or similar (Table 2). Note that R^2 values were greater for the GWR model than for the SLM at both resolutions (Table 2), with the difference being more pronounced at R_{30m} (R^2 of total CO_2 emissions increased almost 0.4) than at R_{500m} (R^2 of total t CO_2 emissions increased almost 0.2). These results indicated that the GWR model was more appropriate than the SLM for describing the relationship between CO_2 emissions and impact factors.

In terms of Fig. 6 a) and d), at R_{30m} the ranges of variation of regression coefficients of all the impact factors were small for MESH. But at R_{500m}, the area with negative regression coefficients was larger than at R_{30m}. Generally, most of the regression coefficients of MESH for four functional land areas were negative (average regression coefficients (ARC) range, −39.43~ −2.35) (Table 3, Appendix A Table S2). However, the industrial and mixed functional land (low-fragmentation level) had positive regression coefficients with CO_2 emissions (ARC range, 1.37–71.94), which was the same positive coefficient at R_{30m} (ARC range, 2.00–30.79). Meanwhile, administrative and public service land (low- and mid-level fragmentation) had mainly negative regression coefficients with CO_2 emissions (ARC range, −8.75~ −0.08) at both R_{30m} and R_{500m}. Besides, residential land with low-level fragmentation also had negative regression coefficients with CO_2 emissions at two resolutions. The common results observed in the low fragmentation level, where the positive relationship between mixed

ARTICLE IN PRESS

Table 2
Comparison between SLM and GWR across resolutions.

CO_2 emission sources	30-m resolution				500-m resolution			
	SLM		GWR		SLM		GWR	
	AIC	R^2	AIC	R^2	AIC	R^2	AIC	R^2
Y_1~X_1+X_2+X_3	649922.1	0.033	607852.1	0.463	5523.6	0.279	5567.1	0.534
Y_2~ X_1+X_2+X_3	382535.3	0.040	314753.6	0.736	4437.0	0.145	4167.9	0.845
Y_3~ X_1+X_2+X_3	648274.6	0.031	605594.4	0.470	5515.9	0.258	5562.0	0.516
Y_4~ X_1+X_2+X_3	139945.6	0.44	113669.0	0.662	3230.1	0.047	3148.5	0.639

*Y_1, Y_2, Y_3, Y_4 represent total, residential, industrial, and transportation CO_2 emissions, respectively. X_1 represents the MESH value of functional district patches. X_2 represents PUA, X_3 represents POID. Abbreviations: SLM, simple linear regression model; GWR, geographically weighted regression model; AIC, Akaike information criterion.

Table 3
The directions of the GWR coefficients for various functional land types and fragmentation levels.

Functional land type	High-MESH (At R_{30m}/R_{500m})	Mid-MESH (At R_{30m}/R_{500m})	Low-MESH (At R_{30m}/R_{500m})
Mixed function and industrial land	+/+	+/−	+/−
Administrative and public service land	−/−	−/−	+/−
Residential land	−/−	+/−	+/−

Note: High-MESH represents low fragmentation. The low fragmentation means MESH = (9.15, 16.32) at R_{30m} and (58.33, 108.33) at R_{500m}.

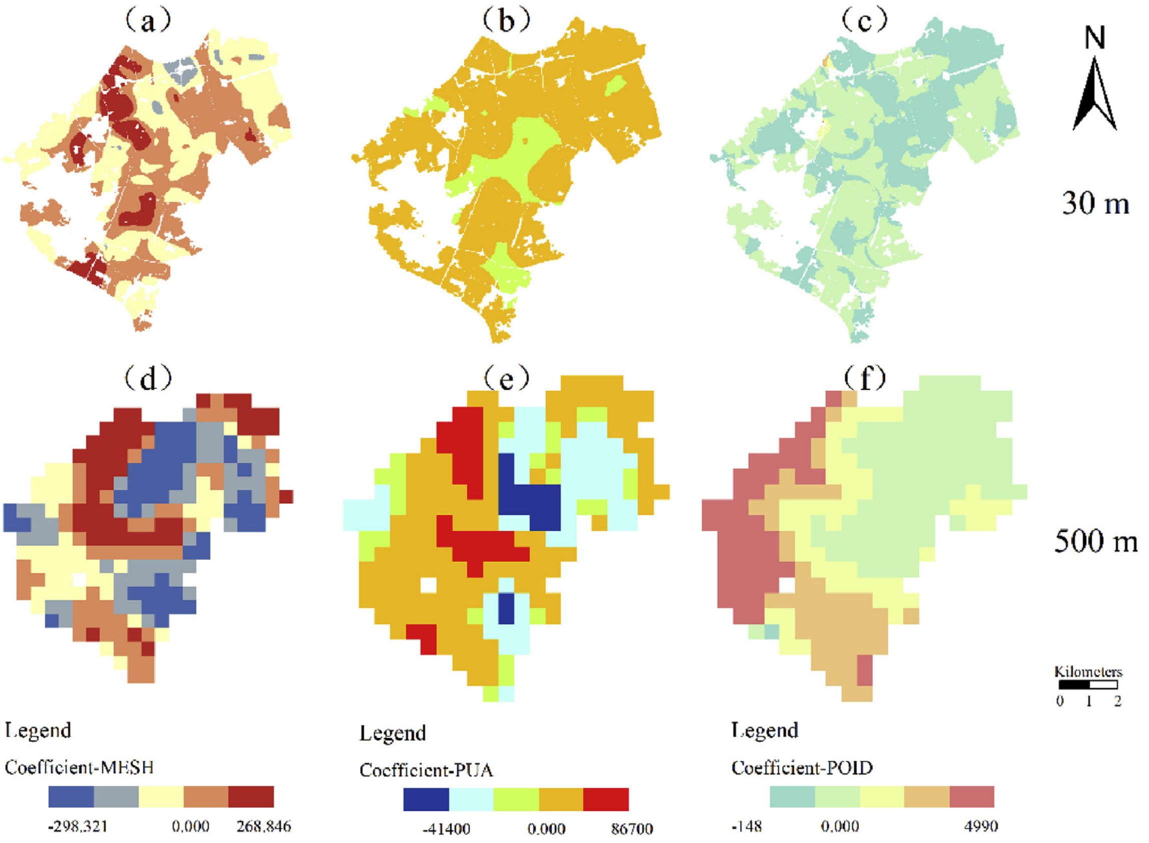

Fig. 6. MESH (a, d), PUA (b, e), and POID (c, f) coefficient estimate maps for total CO_2 emissions in urban functional patches. The R_{30m} maps are shown in the top row (a–c) and the R_{500m} maps are shown in the bottom row (d–f).

functional and industrial areas and the negative relationship between public and residential areas. It implied that mixed functional land and industrial land can be properly dispersed in the central area, but the MESH fragmentation index should be controlled at the ranges of 9.15–16.32 at R_{30m} and 58.33 to 108.33 at R_{500m} based on the classification of the MESH values. The managers should build the contiguous residential and public service land in the low fragmentation area of urban system.

At R_{30m}, PUA and POID, which reflect urban aggregate effects, respectively, had positive regression coefficients with CO_2 emissions, for most of the study area and the absolute values of the regression coefficients were greater than those for MESH. PUA had negative regression coefficients coincident with low-level fragmentation patches near the urban system center. Negative regression coefficients for POID were mostly in mixed function and industrial land areas. The regression coefficient variation range for

Please cite this article as: Zuo, S et al., More fragmentized urban form more CO_2 emissions? A comprehensive relationship from the combination analysis across different scales, Journal of Cleaner Production, https://doi.org/10.1016/j.jclepro.2019.118659

all of the impact factors was an order of magnitude larger at R_{500m} than at R_{30m}.

At R_{500m}, the negative regression coefficient areas for MESH were generally located centrally, coincident with the areas of negative PUA correlation (blue parts in Fig. 6 (e)). It demonstrated that the center of urban system had high PUA, low fragmentation and CO_2 emissions. More than half of the area had positive PUA and POID regression coefficients. The areas with negative PUA and negative MESH regression coefficients were similar. Most of the POID regression coefficients were positive. The areas with small POID regression coefficients often had high-density POIs, with the big POID regression coefficients, the POID was low.

4.3.3. Geographical detector

Geographical detector results at R_{30m} (Fig. 7 a)) showed that the impacts of fragmentation indicators, PUA and POID of urban functional landscape upon CO_2 emissions from different sources were weak, with the exception of the impact of PUA on transportation CO_2 emissions (q value, 0.42). At R_{500m}, the contributions of some urban functional landscape fragmentation indicators of CO_2 emissions from different sources increased, relative those at R_{30m}, moreover the contributions of PUA (q value, 0.13) and POID (q value, 0.3) upon total and industrial CO_2 emissions increased significantly.

In general, the interactions among impact factors affecting CO_2 emissions from different sources were weaker at R_{30m} than at R_{500m} (Fig. 7 b) and c)). We observed three types of interactions of all of the factors including non-linear enhancements ($A \cap B > A + B$), linear enhancements [$A + B > A \cap B > \max(A, B)$], and non-linear weakening [$A \cap B < \min(A, B)$].At R_{30m}, the linear enhancements [$A + B > A \cap B > \max(A, B)$] and non-linear weakening [$A \cap B < \min(A, B)$] accounted for almost half of all of the interactions observed. The factors represent the aggregation effect (PUA and POID) interactions were non-linear enhancements (including CO_2 emissions from all sources).

In terms of combinations of interactions, the trends for interactions for total and industrial CO_2 emissions, were consistent with each other across factors. The interactions among fragmentation factors (NP, PD, DIVISION, MESH) and POID were non-linear enhancements (increase > 0). For combinations of interactions involving residential and transportation CO_2 emissions, there were more factor interactions tended to be linear or non-linear enhancements. For example, besides the interactions of the fragmentation factors with POID that were non-linear enhancements, interactions of fragmentation factors (NP, PD) with PUA were non-linear enhancements (q values > 0.42). Most of the interactions among fragmentation factors were weak, with the exception of the interactions of NP∩DIVISION and PD∩DIVISION for residential CO_2 emissions.

At R_{500m}, most of the interactions among factors impacting CO_2 emissions from different sources showed a strengthening trend. All of the interactions involving CO_2 emissions from different sources were enhancements (linear or non-linear). PUA and POID interactions were weaker at R_{500m} than at R_{30m}.

For combinations of interactions involving total and industrial production CO_2 emissions, the trends were consistent with each other across factors. For example, the interactions of fragmentation factors, such as NP∩MESH and NP∩DIVISION, which represented non-linear enhancements, were more than 100% of the sum of two factors. Although the each factor that influenced residential and transportation CO_2 emissions was weak, we did observe strong interactions of two impact factors. Specifically, the contributions of MESH∩PUA and DIVISION∩PUA to residential CO_2 emissions exceeded 0.14, making them stronger than the impact of the MESH and DIVISION factors alone (without interactions), these interactions were robust non-linear enhancements. MESH∩PUA and DIVISION∩PUA also had strong nonlinear enhancement effects on transportation CO_2 emissions (q values > 0.1).

In summary, the combinations of non-linear interactions of all of the analyzed impact factors differed between the R_{500m} and R_{30m} datasets. The trends of the interactions among the fragmentation factors differed across these two resolutions, most of the interactions of fragmentation factors (especially the total and industrial CO_2 emissions) or the interactions of fragmentation factors and PUA, POID (especially the residential and transportation CO_2 emissions) were weaker at R_{30m} than at $R_{500m,}$ with the exception of the PUA∩POID.

5. Discussion

This study provided a detailed description of the urban functional area using multi-source data. We quantified the intensity and direction of the influencing factors in a stepwise manner, along with the interaction between the two factors at the pixel scale.

5.1. Impacts of scale effect on fragmentation metrics

This study obtained similar results to Saura, with NP and DIVISION showing lower fragmentation at coarser spatial resolutions (Saura, 2004). Since NP is a poor indicator of pattern fragmentation for spatial data lacking exactly the same spatial resolution (Saura, 2002). The low sensitivities of DIVISION and MESH to resolution (grain size) were the consequence of the lower weight given to smaller patches compared to bigger ones. Although some studies use spatial autocorrelation analysis to enrich the landscape pattern studies. We did not use spatial autocorrelation (e.g., Getis statistics, or local Morans I) to characterize landscape fragmentation patterns, because it does not necessarily have theoretical significance. Fan and Myint argued that there is a clear relationship between the autocorrelation index and the landscape fragmentation index, which could be used as a valid exponent representing the heterogeneity of the landscape pattern (Fan and Myint, 2014). However, when considering the actual representation of the degree of aggregation of the patch attributes (continuous variables) in a certain search range, this parameter cannot describe the level of patch fragmentation. These indices could also be considered whether the attribute values of the surrounding patches and those of the targeted patch are similar. Similar values are considered to be aggregated; if patches are not similar, they are not aggregated. This interpretation differs to the principle of the fragmentation index, which needs to reflect the difference between patch types. Even if patch types are numerically assigned, this approach does not indicate whether numerically close patches belong to the same type. However, this type of index has advantages. Because errors in the patch classification phase could be avoided.

Many studies have investigated the importance of scale, using the landscape index to investigate the relationship between landscape pattern and ecological processes, or as an indicator of ecological condition and risk (Luck and Wu, 2002). The results of three methods, namely Pearson correlation analysis, GWR, and geographical detector analyses, had inverted trends and overestimations of single landscape metric factors at R_{500m} compared with results obtained at R_{30m}. Likewise, we observed weaker interactions at R_{30m} than at R_{500m}. Some scholars have indicated that a finer scale results in weaker correlations (Torres et al., 2016), similar to our results. Analysis of the relationship between the two scales showed that GWR and geographical detector analyses (R^2 and q values) produced larger values, that is stronger correlations, at R_{500m} than at R_{30m}, potentially due to changes of interactions among the relative factors. Torres et al. argued that the relationship

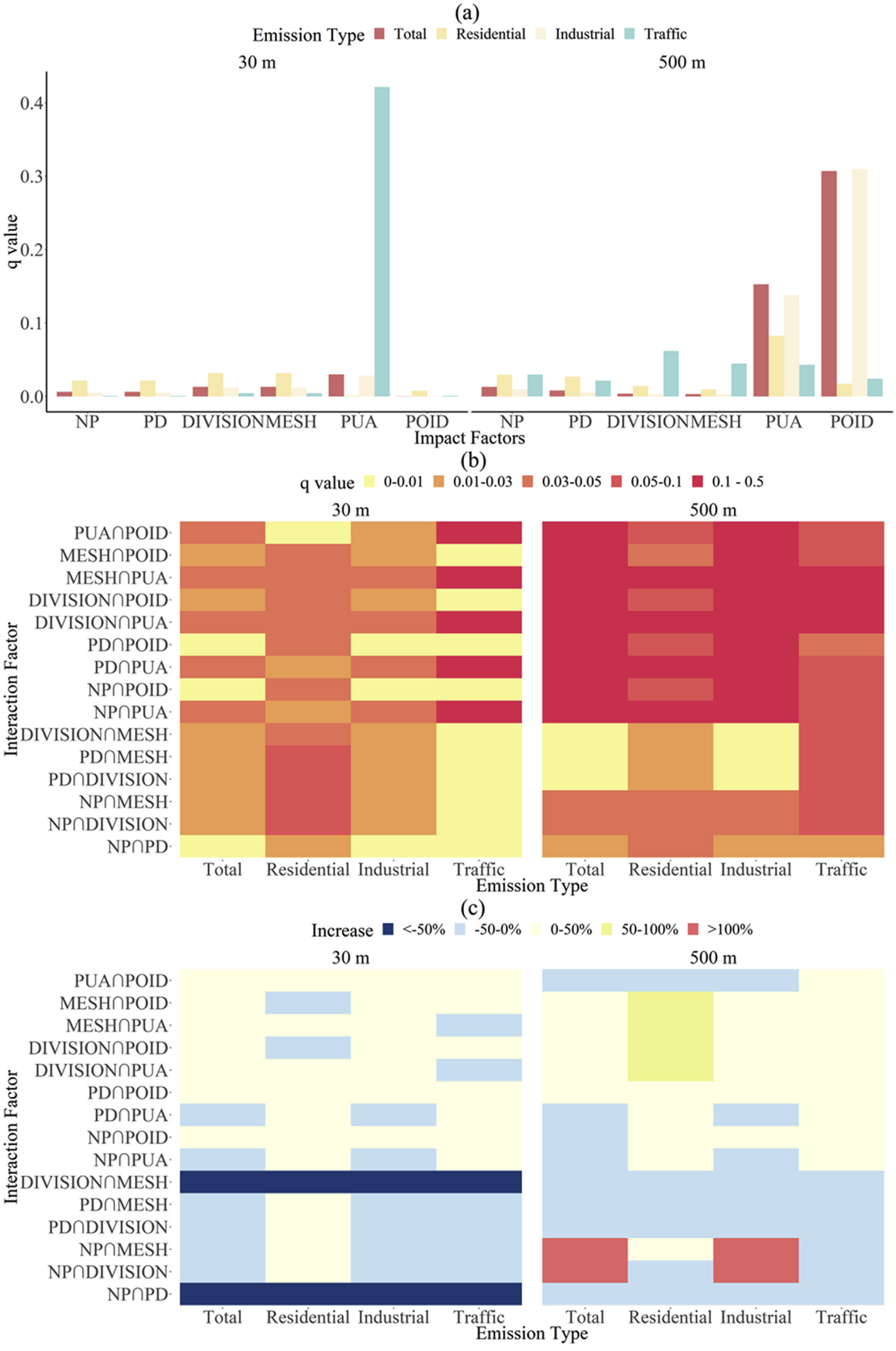

Fig. 7. Geographical detector results for fragmentation factors, PUA, and POID for CO_2 emissions from 4 sources. Residential, industrial, and traffic represent the residential life CO_2 emissions produced by residential living, industrial production, and transportation, respectively. Total represents all CO_2 emissions, that is residential, industrial, and traffic emissions combined. a) The q values of each impact factors including fragmentation factors (NP, PD, DIVISION, MESH), PUA and POID at R_{30m} and R_{500m}; b) The q values of

Please cite this article as: Zuo, S et al., More fragmentized urban form more CO_2 emissions? A comprehensive relationship from the combination analysis across different scales, Journal of Cleaner Production, https://doi.org/10.1016/j.jclepro.2019.118659

between “expansion-fragmentation” differed at different scales, even though the explanatory power of urban expansion on landscape fragmentation is not strong (Torres et al., 2016).

5.2. Relationship between urban fragmentation and CO_2 emissions

Comparing the results obtained from three analytical methods across different spatial resolutions, we confirmed that landscape fragmentation metric correlations with total CO_2 emissions were more significant at R_{500m} than at R_{30m}. Scatter plots of the study area for all grid values were plotted at both scales (Appendix B, Fig. S1). Although we observed similar overall patterns of landscape fragmentation index values across the two scales, the R_{30m} grids had weaker Pearson correlations than were observed at R_{500m}. Yet, both the functional district patches exhibited the opposite phenomenon between the R_{500m} and R_{30m} scales, possibly due to a difference in grain size, with the main type of grid produced at R_{500m} being replaced by other types of grids at R_{30m}. Consequently, areas with high levels of fragmentation at R_{30m} may be found to have less fragmentation at R_{500m}. Alternatively, a grid area that would be classified as non-fragmented at R_{500} may emerge as fragmented at R_{30m}.

At different spatial resolutions, the current study explored the linear relationship between urban morphology and carbon emissions, in addition to focusing on nonlinear direct and interactive relationships. Comparison to the results at the 30-m scale, fragmentation at the 500-m scale had a bigger influence on the patterns of carbon emissions of different functional areas in the city. At R_{30m}, the interactions of fragmentation factors had less impact on CO_2 emission data than the factors without interactions. Hence, there was less spatial similarity of the factors after the interaction than the factors without interactions. This effect might be due to the larger spatial variation of a finer resolution map (Dale et al., 1988). We found that, sometimes, interactions of fragmentation factors that were weak at R_{30m} were relatively stronger R_{500m}, perhaps due to spatial smoothing on the coarser scale.

As to the direction and strength of impact factors, the factors representing urban aggregation effects were more impactful on CO_2 emission data than the landscape fragmentation factor MESH. After controlling for urban aggregation effects, the regression coefficients of MESH showed that fragmentation had positive effects on CO_2 emissions from residential, administrative, and public service (low-level fragmentation) lands and negative effects on CO_2 emissions from mixed function and industrial (low-level fragmentation) lands. These results may be consequent to the large numbers of green belts and plazas near highly fragmented residential land areas, such that more fragmented areas had relatively high emissions. Moreover, administrative and public services lands had low emissions, usually with low fragmentation. As to positive coefficient, mixed function lands were common in the vicinity of the urban center, with numerous mosaic patches of different land use, wherein greater fragmentation was associated with low emissions. Industrial lands with low-level fragmentation were often big-scale production and dispersed industrial emission sources, these industrial emission sources had high emissions with low fragmentation. Notwithstanding, it is important to point out that differences between nighttime light brightness and actual human activities may lead to CO_2 emission mapping errors, and the error would be larger in finer resolution maps (Raupach et al., 2010; Shi et al., 2016). However, areas in which MESH correlated negatively with CO_2 emissions were coincident with low-fragmentation areas, especially in mixed function land areas, because mixed function land characteristics result in a relative neutralization of high and low emissions in other land areas. More to the point, these land areas had low emissions while being located within a central area in the urban system. Therefore, the mixed function zones and industrial parcels with diverse types of land, and the contiguous residential and public service land could correlate with lower emissions in the low fragmentation area of urban system.

In general, we found that interactions between fragmentation parameters and urban aggregation metrics enhanced our ability to explain carbon emission patterns in residential sectors. Consistent with previous studies, we found that urban fragmentation influenced total CO_2 emissions mainly through effects on residential and transportation CO_2 emissions (Creutzig et al., 2015), similar to the relationship between urban landscape fragmentation and urban sprawl. Torres et al. argued that the factors related to urban expansion and landscape fragmentation include aggregation effect, time-lags, spatial arrangements and external variables (topography, teleconnections) (Torres et al., 2016). These factors are not causal relationships, but have a cycle of feedback-promoting mechanisms.

5.3. Significance and limitation

This study showed that high emissions were centered on built-up areas, especially areas with industrial functions. Unlike the pattern observed in very big cities, we found that industrial land areas within the present study area had the most substantial carbon emission levels, while residential districts and transportation areas contributed the least (Xia et al., 2017). This difference is due, presumably, to differences in economic development stage. The main source of income in the present study area is currently agriculture and industry, whereas larger cities have more advanced urban economic development and greater morphological complexity.

Studies of urban form have shown that major metropolitan areas have a mature urban form, whereas relatively lower density, small and medium-sized cities do not. Suburban and exurban areas develop around a central area, with fragmentation and carbon emissions increasing rapidly in exurban areas. Because exurban and central urban areas are the main causes of environmental problems, adjustments should be made when the city is in the state of “suburb” (Xia et al., 2017).

Medium-sized cities share characteristics with suburban and exurban areas of major metropolises, while being less likely to transform into central metropolitan areas because of their geographical locations or resource constraints. Industry and agriculture will remain the main economic engines of the study region for many years to come. However, continued economic development and improvements in living standards are expected to increase traffic and residential emissions. For small and medium-sized cities currently in the initial stages of urban development, transportation emissions are lower than residential emissions. However, future urbanization of currently “exburb” state can bring substantial increases in transportation emissions. By controlling for urban scale aggregate effect factors and selecting appropriate urban system border, our methods have provided an analytic framework for future low-carbon city construction. Our results can be considered representative and universal with respect to similarly

interactions of two impact factors, the combinations of interactions (from bottom to up) were the interactions of different fragmentation factors, the interactions of fragmentation factors, PUA and POID, the interactions of PUA and POID; and c) The increment level of two impact factors. “Increase” means the comparison between the q value of interactions and the sum of two impact factors. “Increase < -50%” means the non-linear weakening, “-50% < Increase <0” means linear enhancements, “0 < Increase<50%” means low-level non-linear enhancement, “50% < Increase<100%” means mid-level non-linear enhancement, “Increase>100%” means high-level non-linear enhancement.

Please cite this article as: Zuo, S et al., More fragmentized urban form more CO_2 emissions? A comprehensive relationship from the combination analysis across different scales, Journal of Cleaner Production, https://doi.org/10.1016/j.jclepro.2019.118659

ARTICLE IN PRESS

situated medium-sized cities.

However, the limitations of our study include the unknown feasibility of the conclusions to mega cities as well as our use of only two resolution scales, which limits our ability to make conclusions regarding a scale effect. Additionally, we didn't examine the impact of socioeconomic factors, such as income and education. In future studies, we plan to address these limitations and to apply the same analytic framework to additional case studies of urban agglomerations and megacities, and to analyze the influence of UFDs on CO_2 emissions across multiple resolutions.

5.4. Spatial resolution selection

Scale selection has long been a problem for landscape ecologists (Plotnick et al., 1993; Wiens, 1989; Wu et al., 2006). The two different resolution data used in our research showed that fragmentation-CO_2 emission results can differ dramatically depending upon spatial resolution, including apparently opposite patterns of results. Even so, some results were consistent across resolution scales. For example, we found that urban aggregation effects were stronger than fragmentation factors (MESH) and that fragmentation correlated positively with lowly fragmented residential, administrative, and public services land use in both the R_{500m} and R_{30m} results. To some extent, the analyses of higher resolution data leads to the comparable but not the same result as the previous studies.

In discussing errors in landscape indexes associated with relatively fine resolution data, scholars that have compared the accuracy of landscape indexes have found that the fitting of an accurate equation was not a good indicator with which to evaluate accuracy and that the variation among different landscape indexes was large and hard to predict (Argañaraz and Entraigas, 2014; Frazier, 2016). Consequently, researchers have developed landscape indexes that are not highly sensitive to spatial resolution. For example, the weighted aggregate contiguity index was developed as a measure of patch compactness and used to reduce errors in analytical results (Zhou et al., 2016). Statistical methods can be used to identify at which resolution analyzed parameter relationships best represent reality. It is important to identify the characteristic scale that provides realistic outcome by modeling the relationship between dependent and independent variables of interest, and thereby determining the scale at which the dependent variable is most strongly affected by the independent variables (Hamil et al., 2016). The main methods used for testing scale suitability have included general linear model (e.g. logistic regression) which is widely used in habitat modeling, machine learning method (e.g. random forest algorithm), geostatistics (e.g. GWR) because of the spatial heterogeneity impacts, and mixed-effect modeling which is suitable for the unknown sources of heterogeneity variables (Graf et al., 2005; Bradter et al., 2013; Li et al., 2017; Hamil et al., 2016). Generally, when conducting research into the relationship between landscape patterns and environmental processes, we should use datasets across multiple spatial scales and combined the background knowledge to make the inferences about the pattern, phenomena and process (Wu, 2004; Wakefield and Lyons, 2010; Li et al., 2015). In this way, results with consistent trends and correlations across different resolutions can be validated, while results that are contradictory across scale levels can be identified and flagged for closer examination. For example, some scholars examined the relationship among multiple ecosystem services. They synthesized cross-scale patterns into a typology of factors could drive changes in ecosystem service relationships across scales (Bennett et al., 2009). Qiu et al. (2018) proposed possible explanations of why the relationships varied across-scale in the consideration of the biophysical connections, interaction among dominant drivers and artificial scale effects. The artificial scale effects which refer to simple alterations to spatial resolution and/or extent of analysis was the case we talk about in this study. The diverse cross-scale patterns we observed needs the further comprehensive analysis and explanations. However, the robust relationships cross the scales suggested the potential efficient management practices. The unstable relationships might signify that the field-specific interventions were insufficient at the landscape scale (Ahiablame et al., 2012).

6. Conclusions

Parallel analyses across two different spatial resolutions, R_{30m} and R_{500m}, showed that fragmentation of urban functional landscape areas in the target urban system appeared to be greater at R_{30m} than at R_{500m}. Within the same resolution scale, fragmentation indicators showed sometimes convergent and sometimes divergent patterns. Secondly, although fragmentation factors influenced urban CO_2 emissions, the impacts of their influences differed with respect to analysis resolution, spatial distribution, and effect power and/or directionality. At R_{30m}, fragmentation factors were found to have weak positive correlations with CO_2 emissions from different sources, whereas at R_{500m}, some of the results were inverted. Thirdly, the GWR results showed that while urban functional landscape fragmentation factors contributed to CO_2 emissions, urban aggregation effects had more potent impacts than fragmentation factors on CO_2 emissions. The government should disperse the mixed function zones and industrial parcels with diverse types land in urban system, and build the contiguous residential and public service land in the low fragmentation area of urban system Finally, the interactions of factors impacting CO_2 emissions from different sources were weaker at R_{30m} than at R_{500m}.

This work makes three main contributions. First, we employ a high-resolution energy-related CO_2 emissions grid mapping methodology to provide fundamental data in support of urban form optimization. Second, we use a spatial analytic framework that includes GWR and geographical detector to explore the relationships between them (linear and non-linear, direct and indirect). We discuss how CO_2 emissions relate to different sources and fragmentation, and thereby clarify the influence of fragmentation of urban functional landscape forms on CO_2 emissions. Third, our conclusions can provide a reference for the study of small and medium coastal towns and cities in China, city management and the planning of low carbon cities. Our analytic framework and multi-source analytic methods are suitable for urban CO_2 emissions studies of agglomerations, mega, small and medium cities. The presently examined study area is a typical of coastal small towns and cities in southeastern China. The central urban system, which accounted for about 10% of the administrative area, produced about 30% of the CO_2 emissions for the whole area. The proportions of CO_2 emission sources in the urban system resembled those for the total city, with industrial, residential, and transportation emissions accounting for >80%, 10—20%, and ~2% of the total emissions.

Acknowledgments

This work was supported by the National Key Research Program of China [grant number 2016YFC0502704]; National Science Foundation of China [grant number 31670645; 31470578; 31200363]; National Social Science Fund [17ZDA058]; Fujian Provincial Science and Technology Project [grant number 2018T3018 and 2015Y0083].

Please cite this article as: Zuo, S et al., More fragmentized urban form more CO_2 emissions? A comprehensive relationship from the combination analysis across different scales, Journal of Cleaner Production, https://doi.org/10.1016/j.jclepro.2019.118659

ARTICLE IN PRESS

Appendix A. Supplementary data

Supplementary data to this article can be found online at https://doi.org/10.1016/j.jclepro.2019.118659.

References

Ahiablame, L.M., Engel, B.A., Chaubey, I., 2012. Effectiveness of low impact development practices: literature review and suggestions for future research. Water Air Soil Pollut. 223, 4253–4273.
Argañaraz, J.P., Entraigas, I., 2014. Scaling functions evaluation for estimation of landscape metrics at higher resolutions. Ecol. Inf. 22, 1–12.
Bennett, E.M., Peterson, G.D., Gordon, L.J., 2009. Understanding relationships among multiple ecosystem services. Ecol. Lett. 12, 1394–1404.
Bitter, C., Mulligan, G.F., Dall'erba, S., 2007. Incorporating spatial variation in housing attribute prices: a comparison of geographically weighted regression and the spatial expansion method. J. Geogr. Syst. 9, 7–27.
Bradter, U., Kunin, W.E., Altringham, J.D., Thom, T.J., Benton, T.G., 2013. Identifying appropriate spatial scales of predictors in species distribution models with the random forest algorithm. Methods Evol. Ecol. 4, 167–174.
Brunsdon, C., Fotheringham, S., Charlton, M., 1998. Geographically weighted regression—modelling spatial non-stationarity. J. R. Stat. Soc. - Ser. D Statistician 47, 431–443.
Cahill, M., Mulligan, G., 2007. Using geographically weighted regression to explore local crime patterns. Soc. Sci. Comput. Rev. 25, 174–193.
Cai, B., Zhang, L., 2014. Urban CO2 emissions in China: spatial boundary and performance comparison. Energy Policy 66, 557–567.
Cai, J., Huang, B., Song, Y., 2017. Using multi-source geospatial big data to identify the structure of polycentric cities. Remote Sens. Environ. 202, 210–221.
Christen, A., Coops, N.C., Crawford, B.R., Kellett, R., Liss, K.N., Olchovski, I., et al., 2011. Validation of modeled carbon-dioxide emissions from an urban neighborhood with direct eddy-covariance measurements. Atmos. Environ. 45, 6057–6069.
Creutzig, F., Jochem, P., Edelenbosch, O.Y., Mattauch, L., DPv, Vuuren, McCollum, D., et al., 2015. Transport: a roadblock to climate change mitigation? Science 350, 911–912.
Dai, S.Q., Zuo, S.D., Ren, Y., 2019. A Spatial Database of CO_2 Emission and Urban Form Fragmentation for the Low Carbon Urban System in Jinjiang City, China. Data in Brief.
Dale, V.H., Jager, H.I., Gardner, R.H., Rosen, A.E., 1988. Using sensitivity and uncertainty analyses to improve predictions of broad-scale forest development. Ecol. Model. 42, 165–178.
Fan, C., Myint, S., 2014. A comparison of spatial autocorrelation indices and landscape metrics in measuring urban landscape fragmentation. Landsc. Urban Plan. 121, 117–128.
Fang, C., Wang, S., Li, G., 2015. Changing urban forms and carbon dioxide emissions in China: a case study of 30 provincial capital cities. Appl. Energy 158, 519–531.
Frazier, A.E., 2016. Surface metrics: scaling relationships and downscaling behavior. Landsc. Ecol. 31, 351–363.
Gao, J., Li, S., 2011. Detecting spatially non-stationary and scale-dependent relationships between urban landscape fragmentation and related factors using Geographically Weighted Regression. Appl. Geogr. 31, 292–302.
Gately, C.K., Hutyra, L.R., 2017. Large uncertainties in urban-scale carbon emissions. J. Geophys. Res.: Atmospheres 11, 242–260. https://doi.org/10.1002/2017JD027359.
Girvetz, E.H., Thorne, J.H., Berry, A.M., Jaeger, J.A.G., 2008. Integration of landscape fragmentation analysis into regional planning: a statewide multi-scale case study from California, USA. Landsc. Urban Plan. 86, 205–218.
Gong, C., Yu, S., Joesting, H., Chen, J., 2013. Determining socioeconomic drivers of urban forest fragmentation with historical remote sensing images. Landsc. Urban Plan. 117, 57–65.
Graf, R.F., Bollmann, K., Suter, W., Bugmann, H., 2005. The importance of spatial scale in habitat models: capercaillie in the Swiss Alps. Landsc. Ecol. 20, 703–717.
Hamil, K.-A.D., Iannone III, B.V., Huang, W.K., Fei, S., Zhang, H., 2016. Cross-scale contradictions in ecological relationships. Landsc. Ecol. 31, 7–18.
Hartigan, J.A., Wong, M.A., 1979. Algorithm as 136: a K-means clustering algorithm. J. R. Stat. Soc. Ser. C Appl. Stat. 28 (1), 100–108.
He, Z., Xu, S., Shen, W., Long, R., Chen, H., 2017. Impact of urbanization on energy related CO2 emission at different development levels: regional difference in China based on panel estimation. J. Clean. Prod. 140, 1719–1730.
Hu, M.G., Li, Z.J., Wang, J.F., Jia, L., Liao, Y.L., Lai, S.J., et al., 2012. Determinants of the incidence of hand, foot and mouth disease in China using geographically weighted regression models. PLoS One 7.
Hu, X., Waller, L.A., Al-Hamdan, M.Z., Crosson, W.L., Estes, M.G., Estes, S.M., et al., 2013. Estimating ground-level $PM_{2.5}$ concentrations in the southeastern US using Geographically Weighted Regression. Environ. Res. 121, 1–10.
Lee, S., Lee, B., 2014. The influence of urban form on GHG emissions in the U.S. household sector. Energy Policy 68, 534–549.
Li, C., Li, F., Wu, Z., Cheng, J., 2015. Effects of landscape heterogeneity on the elevated trace metal concentrations in agricultural soils at multiple scales in the Pearl River Delta, South China. Environ. Pollut. 206, 264–274.
Li, C., Li, F., Wu, Z., Cheng, J., 2017. Exploring spatially varying and scale-dependent relationships between soil contamination and landscape patterns using geographically weighted regression. Appl. Geogr. 82, 101–114.
Lin, T., Sun, C.G., Li, X.H., Zhao, Q.J., Zhang, G.Q., Ge, R.B., et al., 2016. Spatial pattern of urban functional landscapes along an urban–rural gradient: a case study in Xiamen City, China. Int. J. Appl. Earth Obs. Geoinf. 46, 22–30.
Liu, X., Sweeney, J., 2012. Modelling the impact of urban form on household energy demand and related CO_2 emissions in the Greater Dublin Region. Energy Policy 46, 359–369.
Luck, M., Wu, J.G., 2002. A gradient analysis of urban landscape pattern: a case study from the Phoenix metropolitan region, Arizona, USA. Landsc. Ecol. 17, 327–339.
Nordbo, A., Järvi, L., Haapanala, S., Wood, C.R., Vesala, T., 2012. Fraction of natural area as main predictor of net CO2 emissions from cities. Geophys. Res. Lett. 39.
Ou, J.P., Liu, X.P., Li, X., Chen, Y.M., 2013. Quantifying the relationship between urban forms and carbon emissions using panel data analysis. Landsc. Ecol. 28, 1889–1907.
Owens, S.E., 1986. Energy, Planning and Urban Form. Taylor & Francis.
Plotnick, R.E., Gardner, R.H., Oneill, R.V., 1993. Lacunarity indexes as measures of landscape texture. Landsc. Ecol. 8, 201–211.
Qiu, J.X., Carpenter, S.R., Booth, E.G., Motew, M., Zipper, S.C., Kucharik, C.J., et al., 2018. Understanding relationships among ecosystem services across spatial scales and over time. Environ. Res. Lett. 13, 054020.
Raupach, M.R., Rayner, P.J., Paget, M., 2010. Regional variations in spatial structure of nightlights, population density and fossil-fuel CO_2 emissions. Energy Policy 38, 4756–4764.
Ren, Y., Deng, L.Y., Zuo, S.D., Song, X.D., Liao, Y.L., Xu, C.D., et al., 2016. Quantifying the influences of various ecological factors on land surface temperature of urban forests. Environ. Pollut. 216, 519–529.
Saura, S., 2002. Effects of minimum mapping unit on land cover data spatial configuration and composition. Int. J. Remote Sens. 23, 4853–4880.
Saura, S., 2004. Effects of remote sensor spatial resolution and data aggregation on selected fragmentation indices. Landsc. Ecol. 19, 197–209.
Shi, K.F., Chen, Y., Yu, B.L., Xu, T.B., Chen, Z.Q., Liu, R., et al., 2016. Modeling spatio-temporal CO_2 (carbon dioxide) emission dynamics in China from DMSP-OLS nighttime stable light data using panel data analysis. Appl. Energy 168, 523–533.
Torres, A., Jaeger, J.A.G., Alonso, J.C., 2016. Multi-scale mismatches between urban sprawl and landscape fragmentation create windows of opportunity for conservation development. Landsc. Ecol. 31, 2291–2305.
Wakefield, J., Lyons, H., 2010. Spatial aggregation and the ecological fallacy. In: Handbook of Spatial Statistics. CRC Press, pp. 537–554.
Wang, J.F., Li, X.H., Christakos, G., Liao, Y.L., Zhang, T., Gu, X., et al., 2010. Geographical Detectors-based health risk assessment and its application in the neural tube defects study of the Heshun region, China. Int. J. Geogr. Inf. Sci. 24, 107–127.
Wang, J.F., Zhang, T.L., Fu, B.J., 2016a. A measure of spatial stratified heterogeneity. Ecol. Indicat. 67, 250–256.
Wang, Q., Ni, J., Tenhunen, J., 2005. Application of a geographically-weighted regression analysis to estimate net primary production of Chinese forest ecosystems. Glob. Ecol. Biogeogr. 14, 379–393.
Wang, S., Fang, C., Guan, X., Pang, B., Ma, H., 2014. Urbanisation, energy consumption, and carbon dioxide emissions in China: a panel data analysis of China's provinces. Appl. Energy 136, 738–749.
Wang, S., Fang, C., Wang, Y., 2016b. Spatiotemporal variations of energy-related CO_2 emissions in China and its influencing factors: an empirical analysis based on provincial panel data. Renew. Sustain. Energy Rev. 55, 505–515.
Wang, S., Liu, X., Zhou, C., Hu, J., Ou, J., 2017. Examining the impacts of socioeconomic factors, urban form, and transportation networks on CO_2 emissions in China's megacities. Appl. Energy 185, 189–200.
Wang, S., Zhou, C., Li, G., Feng, K., 2016c. CO2, economic growth, and energy consumption in China's provinces: investigating the spatiotemporal and econometric characteristics of China's CO_2 emissions. Ecol. Indicat. 69, 184–195.
Wang, S.J., Liu, X.P., 2017. China's city-level energy-related CO2 emissions: spatiotemporal patterns and driving forces. Appl. Energy 200, 204–214.
Wang, S., Li, G., Fang, C., 2018b. Urbanization, economic growth, energy consumption, and CO2 emissions: Empirical evidence from countries with different income levels. Renew. Sust. Energ. Rev. 81, 2144–2159.
Wang, Y., Li, L., Kubota, J., Han, R., Zhu, X., Lu, G., 2016d. Does urbanization lead to more carbon emission? Evidence from a panel of BRICS countries. Appl. Energy 168, 375–380.
Wang, Z., Yin, F., Zhang, Y., Zhang, X., 2012. An empirical research on the influencing factors of regional CO_2 emissions: evidence from Beijing city, China. Appl. Energy 100, 277–284.
Wiens, J.A., 1989. Spatial scaling in ecology. Funct. Ecol. 3, 385–397.
Wu, J., Jones, B., Li, H., Loucks, O.L., 2006. Scaling and Uncertainty Analysis in Ecology. Springer.
Wu, J.G., 2004. Effects of changing scale on landscape pattern analysis: scaling relations. Landsc. Ecol. 19, 125–138.
Xia, L.L., Zhang, Y., Sun, X.X., Li, J.J., 2017. Analyzing the spatial pattern of carbon metabolism and its response to change of urban form. Ecol. Model. 355, 105–115.
Ye, H., He, X.Y., Song, Y., Li, X.H., Zhang, G.Q., Lin, T., et al., 2015. A sustainable urban form: the challenges of compactness from the viewpoint of energy consumption and carbon emission. Energy Build. 93, 90–98.
Zhang, N., Yu, K., Chen, Z., 2017. How does urbanization affect carbon dioxide emissions? A cross-country panel data analysis. Energy Policy 107, 678–687.
Zhou, J., Xiao, N., Liu, L., Li, Q., 2016. A weighted aggregation and closeness approach to measuring the compactness of landscape with multiple parts. Ecol. Indicat. 64, 158–170.
