# Supplementary material for: A spatial database of CO2 emissions, urban form fragmentation and city-scale effect related impact factors for the low carbon urban system in Jinjiang city, China
Source: Data Brief. 2020 Feb 11;29:105274. doi: 10.1016/j.dib.2020.105274 (PMC7042417; doi:10.1016/j.dib.2020.105274)
Supplement: Multimedia component 1 [file mmc1.zip › 数据包/presentation/DIBdata.html]

A spatial database of CO\_2 emissions and urban form fragmentation for the low carbon urban system in Jinjiang city, China


# A spatial database of \(CO\_2\) emissions and urban form fragmentation for the low carbon urban system in Jinjiang city, China

- Introduction
- Database
  - Spatial distribution maps of \(CO\_2\) emissions
    - 30 m resolution maps
    - 500 m resolution maps
  - The mixing degree of urban functional district (UFD)
  - Lacunarity indices
  - Landscape metrics
    - 30 m resolution maps
    - 500 m resolution maps
  - Impact factors of the \(CO\_2\) mitigation: PUA and POID
    - PUA
    - POID
- Reference

# Introduction

A spatial database of low carbon urban system represented the spatial distribution maps of \(CO\_2\) emissions, urban form metrics (urban landscape fragmentation), proportion of urban area (PUA) and points of interest density (POID) at two resolutions: 30 m (\(R\_{30m}\)) and 500 m (\(R\_{500m}\)) in Jinjiang city, China. The data were produced from ArcGIS 10.2, Apack 2.23, Fragstats 4.2 and R 3.5.3. All the data were stored in Geotiff format.

The names ofcorresponding spatial data of Geotiff files given in the ReadMe.txt.

# Database

## Spatial distribution maps of \(CO\_2\) emissions

### 30 m resolution maps

Download links as follow:

“Total”, “Resident”, “Industry” and “Transport” represent the total, resident, industrial, and Transport \(CO\_2\) emissions in Jinjiang City respectively.

Total

Resident

Industry

Transport

### 500 m resolution maps

Download links as follow:

“Total”, “Resident”, “Industry” and “Transport” represent the total, resident, industrial, and Transport \(CO\_2\) emissions in Jinjiang City respectively.

Total

Resident

Industry

Transport

## The mixing degree of urban functional district (UFD)

Download links as follow:

R 30 m R 500 m

## Lacunarity indices

We used Apack 2.23 calculated the Lacunarity indicies.

Download links as follow:

R 30 m R 500 m

## Landscape metrics

Fragstats 4.2 software was used to calculate the number of patches (NP), patch density (PD), division (DIVISION) and effective mesh size (MESH) metrics.

### 30 m resolution maps

Download links as follow:

NP

PD

DIVISION

MESH

### 500 m resolution maps

Download links as follow:

NP

PD

DIVISION

MESH

## Impact factors of the \(CO\_2\) mitigation: PUA and POID

### PUA

Download links as follow:

R 30 m

R 500 m

### POID

Download links as follow:

R 30 m

R 500 m

# Reference

If you used the data[1,2], please cited as follow:

[1] Dai S, Zuo S, Ren Y. High-resolution mapping of direct \(CO\_2\) emissions and uncertainties at the urban scale[A]. Spatial Accuracy 2018, May 21, 2018 - May 25, 2018[C]. Aussino Academic Publishing House: 88–90.

[2] Zuo S, Dai S, Ren Y. More fragmentized urban form more \(CO\_2\) emissions? A comprehensive relationship from the combination analysis across different scales[J]. Journal of Cleaner Production, 2019: 118659.
