# Supplementary material for: A spatial database of CO2 emissions, urban form fragmentation and city-scale effect related impact factors for the low carbon urban system in Jinjiang city, China
Source: Data Brief. 2020 Feb 11;29:105274. doi: 10.1016/j.dib.2020.105274 (PMC7042417; doi:10.1016/j.dib.2020.105274)
Supplement: Multimedia component 1 [file mmc1.zip › 数据包/presentation/DIBdata.nb.html]

A spatial database of CO\_2 emissions and urban form fragmentation for the low carbon urban system in Jinjiang city, China


Code 

- Show All Code
- Hide All Code
- Download Rmd

# A spatial database of \(CO\_2\) emissions and urban form fragmentation for the low carbon urban system in Jinjiang city, China

- Introduction
- Database
  - Spatial distribution maps of \(CO\_2\) emissions
    - 30 m resolution maps
    - 500 m resolution maps
  - The mixing degree of urban functional district (UFD)
  - Lacunarity indices
  - Landscape metrics
    - 30 m resolution maps
    - 500 m resolution maps
  - Impact factors of the \(CO\_2\) mitigation: PUA and POID
    - PUA
    - POID
- Reference

# Introduction

A spatial database of low carbon urban system represented the spatial distribution maps of \(CO\_2\) emissions, urban form metrics (urban landscape fragmentation), proportion of urban area (PUA) and points of interest density (POID) at two resolutions: 30 m (\(R\_{30m}\)) and 500 m (\(R\_{500m}\)) in Jinjiang city, China. The data were produced from ArcGIS 10.2, Apack 2.23, Fragstats 4.2 and R 3.5.3. All the data were stored in Geotiff format.

The names ofcorresponding spatial data of Geotiff files given in the ReadMe.txt.

# Database

## Spatial distribution maps of \(CO\_2\) emissions

### 30 m resolution maps


Download links as follow:

“Total”, “Resident”, “Industry” and “Transport” represent the total, resident, industrial, and Transport \(CO\_2\) emissions in Jinjiang City respectively.

Total

Resident

Industry

Transport

### 500 m resolution maps


Download links as follow:

“Total”, “Resident”, “Industry” and “Transport” represent the total, resident, industrial, and Transport \(CO\_2\) emissions in Jinjiang City respectively.

Total

Resident

Industry

Transport

## The mixing degree of urban functional district (UFD)


Download links as follow:

R 30 m R 500 m

## Lacunarity indices

We used Apack 2.23 calculated the Lacunarity indicies.


Download links as follow:

R 30 m R 500 m

## Landscape metrics

Fragstats 4.2 software was used to calculate the number of patches (NP), patch density (PD), division (DIVISION) and effective mesh size (MESH) metrics.

### 30 m resolution maps


Download links as follow:

NP

PD

DIVISION

MESH

### 500 m resolution maps


Download links as follow:

NP

PD

DIVISION

MESH

## Impact factors of the \(CO\_2\) mitigation: PUA and POID

### PUA


Download links as follow:

R 30 m

R 500 m

### POID


Download links as follow:

R 30 m

R 500 m

# Reference

If you used the data[1,2], please cited as follow:

[1] Dai S, Zuo S, Ren Y. High-resolution mapping of direct \(CO\_2\) emissions and uncertainties at the urban scale[A]. Spatial Accuracy 2018, May 21, 2018 - May 25, 2018[C]. Aussino Academic Publishing House: 88–90.

[2] Zuo S, Dai S, Ren Y. More fragmentized urban form more \(CO\_2\) emissions? A comprehensive relationship from the combination analysis across different scales[J]. Journal of Cleaner Production, 2019: 118659.

LS0tDQp0aXRsZTogQSBzcGF0aWFsIGRhdGFiYXNlIG9mICRDT18yJCBlbWlzc2lvbnMgYW5kIHVyYmFuIGZvcm0gZnJhZ21lbnRhdGlvbiBmb3IgdGhlDQogIGxvdyBjYXJib24gdXJiYW4gc3lzdGVtIGluIEppbmppYW5nIGNpdHksIENoaW5hDQpjc2w6IGNoaW5lc2UtZ2I3NzE0LTE5ODctbnVtZXJpYy5jc2wNCm91dHB1dDoNCiAgaHRtbF9kb2N1bWVudDoNCiAgICBkZl9wcmludDogcGFnZWQNCiAgICB0b2M6IHllcw0KICBodG1sX25vdGVib29rOg0KICAgIHRvYzogeWVzDQpiaWJsaW9ncmFwaHk6IENFTVUuYmliDQotLS0NCg0KIyBJbnRyb2R1Y3Rpb24NCg0KQSBzcGF0aWFsIGRhdGFiYXNlIG9mIGxvdyBjYXJib24gdXJiYW4gc3lzdGVtIHJlcHJlc2VudGVkIHRoZSBzcGF0aWFsIGRpc3RyaWJ1dGlvbiBtYXBzIG9mICRDT18yJCBlbWlzc2lvbnMsIHVyYmFuIGZvcm0gbWV0cmljcyAodXJiYW4gbGFuZHNjYXBlIGZyYWdtZW50YXRpb24pLCBwcm9wb3J0aW9uIG9mIHVyYmFuIGFyZWEgKFBVQSkgYW5kIHBvaW50cyBvZiBpbnRlcmVzdCBkZW5zaXR5IChQT0lEKSBhdCB0d28gcmVzb2x1dGlvbnM6IDMwIG0gKCRSX3szMG19JCkgYW5kIDUwMCBtICgkUl97NTAwbX0kKSBpbiBKaW5qaWFuZyBjaXR5LCBDaGluYS4gVGhlIGRhdGEgd2VyZSBwcm9kdWNlZCBmcm9tIEFyY0dJUyAxMC4yLCBBcGFjayAyLjIzLCBGcmFnc3RhdHMgNC4yIGFuZCBSIDMuNS4zLiBBbGwgdGhlIGRhdGEgd2VyZSBzdG9yZWQgaW4gR2VvdGlmZiBmb3JtYXQuDQoNClRoZSBuYW1lcyBvZmNvcnJlc3BvbmRpbmcgc3BhdGlhbCBkYXRhIG9mIEdlb3RpZmYgZmlsZXMgZ2l2ZW4gaW4gdGhlIFtSZWFkTWUudHh0XShodHRwOi8vc2NpZW5jZS5naXNlcnNxZGFpLnRvcC9ESUIvRGF0YURlc2NyaXB0aW9uLnR4dCkuDQoNCiMgRGF0YWJhc2UNCg0KIyMgU3BhdGlhbCBkaXN0cmlidXRpb24gbWFwcyBvZiAkQ09fMiQgZW1pc3Npb25zDQojIyMgMzAgbSByZXNvbHV0aW9uIG1hcHMNCmBgYHtyIGVjaG89RkFMU0UsIG1lc3NhZ2U9RkFMU0UsIHdhcm5pbmc9RkFMU0V9DQpsaWJyYXJ5KHJhc3RlcikNCmxpYnJhcnkobGVhZnN5bmMpDQpsaWJyYXJ5KG1hcHZpZXcpDQoNCkNPUDMwIDwtICByYXN0ZXIoIkY6L0dJU3Byb2plY3QvSVVFL0NhcmJvbkVtaXNzaW9uL0ZpbmFsL1NlY29uZFJlc3VsdC9VbmNlcnRhaW50eS9NYXBwaW5nL0NPUDMwLnRpZiIpDQpDT0kzMCA8LSByYXN0ZXIoIkY6L0dJU3Byb2plY3QvSVVFL0NhcmJvbkVtaXNzaW9uL0ZpbmFsL1NlY29uZFJlc3VsdC9VbmNlcnRhaW50eS9NYXBwaW5nL0NPSTMwLnRpZiIpDQpDT1QzMCA8LSByYXN0ZXIoIkY6L0dJU3Byb2plY3QvSVVFL0NhcmJvbkVtaXNzaW9uL0ZpbmFsL1NlY29uZFJlc3VsdC9VbmNlcnRhaW50eS9NYXBwaW5nL0NPVDMwLnRpZiIpDQpDTzMwIDwtIHJhc3RlcigiRjovR0lTcHJvamVjdC9JVUUvQ2FyYm9uRW1pc3Npb24vRmluYWwvU2Vjb25kUmVzdWx0L1VuY2VydGFpbnR5L01hcHBpbmcvQ08zMC50aWYiKQ0KDQptMSA8LSBtYXB2aWV3KENPMzAsIGxheWVyLm5hbWUgPSAiVG90YWwiKQ0KbTIgPC0gbWFwdmlldyhDT1AzMCwgbGF5ZXIubmFtZSA9ICJSZXNpZGVudCIpDQptMyA8LSBtYXB2aWV3KENPSTMwLCBsYXllci5uYW1lID0gIkluZHVzdHJ5IikNCm00IDwtIG1hcHZpZXcoQ09UMzAsIGxheWVyLm5hbWUgPSAiVHJhbnNwb3J0IikNCnN5bmMobTEsIG0yLCBtMywgbTQpDQpgYGANCg0KRG93bmxvYWQgbGlua3MgYXMgZm9sbG93Og0KDQoiVG90YWwiLCAiUmVzaWRlbnQiLCAiSW5kdXN0cnkiIGFuZCAiVHJhbnNwb3J0IiByZXByZXNlbnQgdGhlIHRvdGFsLCByZXNpZGVudCwgaW5kdXN0cmlhbCwgYW5kIFRyYW5zcG9ydCAkQ09fMiQgZW1pc3Npb25zIGluIEppbmppYW5nIENpdHkgcmVzcGVjdGl2ZWx5LiANCg0KW1RvdGFsXShodHRwOi8vc2NpZW5jZS5naXNlcnNxZGFpLnRvcC9ESUIvQ08zMC50aWYpDQoNCltSZXNpZGVudF0oaHR0cDovL3NjaWVuY2UuZ2lzZXJzcWRhaS50b3AvRElCL0NPUjMwLnRpZikNCg0KW0luZHVzdHJ5XShodHRwOi8vc2NpZW5jZS5naXNlcnNxZGFpLnRvcC9ESUIvQ09JMzAudGlmKQ0KDQpbVHJhbnNwb3J0XShodHRwOi8vc2NpZW5jZS5naXNlcnNxZGFpLnRvcC9ESUIvQ09UMzAudGlmKQ0KDQoNCiMjIyA1MDAgbSByZXNvbHV0aW9uIG1hcHMNCmBgYHtyIGVjaG89RkFMU0UsIG1lc3NhZ2U9RkFMU0UsIHdhcm5pbmc9RkFMU0V9DQpDT1A1MDAgPC0gIHJhc3RlcigiRjovR0lTcHJvamVjdC9JVUUvQ2FyYm9uRW1pc3Npb24vRmluYWwvU2Vjb25kUmVzdWx0L1VuY2VydGFpbnR5L01hcHBpbmcvQ09QNTAwLnRpZiIpDQpDT0k1MDAgPC0gcmFzdGVyKCJGOi9HSVNwcm9qZWN0L0lVRS9DYXJib25FbWlzc2lvbi9GaW5hbC9TZWNvbmRSZXN1bHQvVW5jZXJ0YWludHkvTWFwcGluZy9DT0k1MDAudGlmIikNCkNPVDUwMCA8LSByYXN0ZXIoIkY6L0dJU3Byb2plY3QvSVVFL0NhcmJvbkVtaXNzaW9uL0ZpbmFsL1NlY29uZFJlc3VsdC9VbmNlcnRhaW50eS9NYXBwaW5nL0NPVDUwMC50aWYiKQ0KQ081MDAgPC0gcmFzdGVyKCJGOi9HSVNwcm9qZWN0L0lVRS9DYXJib25FbWlzc2lvbi9GaW5hbC9TZWNvbmRSZXN1bHQvVW5jZXJ0YWludHkvTWFwcGluZy9DTzUwMC50aWYiKQ0KDQptNSA8LSBtYXB2aWV3KENPNTAwLCBsYXllci5uYW1lID0gIlRvdGFsIikNCm02IDwtIG1hcHZpZXcoQ09QNTAwLCBsYXllci5uYW1lID0gIlJlc2lkZW50IikNCm03IDwtIG1hcHZpZXcoQ09JNTAwLCBsYXllci5uYW1lID0gIkluZHVzdHJ5IikNCm04IDwtIG1hcHZpZXcoQ09UNTAwLCBsYXllci5uYW1lID0gIlRyYW5zcG9ydCIpDQoNCnN5bmMobTUsIG02LCBtNywgbTgpDQpgYGANCg0KRG93bmxvYWQgbGlua3MgYXMgZm9sbG93Og0KDQoiVG90YWwiLCAiUmVzaWRlbnQiLCAiSW5kdXN0cnkiIGFuZCAiVHJhbnNwb3J0IiByZXByZXNlbnQgdGhlIHRvdGFsLCByZXNpZGVudCwgaW5kdXN0cmlhbCwgYW5kIFRyYW5zcG9ydCAkQ09fMiQgZW1pc3Npb25zIGluIEppbmppYW5nIENpdHkgcmVzcGVjdGl2ZWx5LiANCg0KW1RvdGFsXShodHRwOi8vc2NpZW5jZS5naXNlcnNxZGFpLnRvcC9ESUIvQ081MDAudGlmKQ0KDQpbUmVzaWRlbnRdKGh0dHA6Ly9zY2llbmNlLmdpc2Vyc3FkYWkudG9wL0RJQi9DT1I1MDAudGlmKQ0KDQpbSW5kdXN0cnldKGh0dHA6Ly9zY2llbmNlLmdpc2Vyc3FkYWkudG9wL0RJQi9DT0k1MDAudGlmKQ0KDQpbVHJhbnNwb3J0XShodHRwOi8vc2NpZW5jZS5naXNlcnNxZGFpLnRvcC9ESUIvQ09UNTAwLnRpZikNCg0KIyMgVGhlIG1peGluZyBkZWdyZWUgb2YgdXJiYW4gZnVuY3Rpb25hbCBkaXN0cmljdCAoVUZEKQ0KDQpgYGB7ciBlY2hvPUZBTFNFLCBtZXNzYWdlPUZBTFNFLCB3YXJuaW5nPUZBTFNFfQ0KZmNtaXgzMCA8LSAgcmFzdGVyKCJGOi9HSVNwcm9qZWN0L0lVRS9DYXJib25FbWlzc2lvbi9GaW5hbC9TZWNvbmRSZXN1bHQvbWl4ZWQvcGxhbm5pbmdmdW5jdGlvbm1peC50aWYiKQ0KZmNtaXg1MDAgPC0gcmFzdGVyKCJGOi9HSVNwcm9qZWN0L0lVRS9DYXJib25FbWlzc2lvbi9GaW5hbC9TZWNvbmRSZXN1bHQvbWl4ZWQvZnVuY3Rpb25taXg1MDBtLnRpZiIpDQoNCm05IDwtIG1hcHZpZXcoZmNtaXgzMCoxMDAsIGxheWVyLm5hbWUgPSAiUiAzMCBtL3BlcmNlbnRhZ2UiKQ0KbTEwIDwtIG1hcHZpZXcoZmNtaXg1MDAqMTAwLCBsYXllci5uYW1lID0gIlIgNTAwIG0vcGVyY2VudGFnZSIpDQoNCnN5bmMobTksIG0xMCkNCmBgYA0KDQpEb3dubG9hZCBsaW5rcyBhcyBmb2xsb3c6DQoNCltSIDMwIG1dKGh0dHA6Ly9zY2llbmNlLmdpc2Vyc3FkYWkudG9wL0RJQi9mY21peDMwLnRpZikNCltSIDUwMCBtXShodHRwOi8vc2NpZW5jZS5naXNlcnNxZGFpLnRvcC9ESUIvZmNtaXg1MDAudGlmKQ0KDQojIyBMYWN1bmFyaXR5IGluZGljZXMNCldlIHVzZWQgQXBhY2sgMi4yMyBjYWxjdWxhdGVkIHRoZSBMYWN1bmFyaXR5IGluZGljaWVzLiANCg0KYGBge3IgZWNobz1GQUxTRSwgZmlnLmhlaWdodD01LCBmaWcud2lkdGg9OCwgbWVzc2FnZT1GQUxTRSwgd2FybmluZz1GQUxTRSwgcGFnZWQucHJpbnQ9RkFMU0V9DQpsaWJyYXJ5KGdncGxvdDIpDQpsaWJyYXJ5KGdndGhlbXIpDQpsaWJyYXJ5KGdyaWRFeHRyYSkNCg0Kd2luZG93c0ZvbnRzKFJUID0gd2luZG93c0ZvbnQoIlRpbWVzIE5ldyBSb21hbiIpKQ0KDQpMQ1U8LXJlYWQudGFibGUoIkY6L0dJU3Byb2plY3QvSVVFL0NhcmJvbkVtaXNzaW9uL0ZpbmFsL1NlY29uZFJlc3VsdC9TY2FsZS9MQ1UudHh0IixzZXA9IiIpDQpsZnR5cGUxPC1jKCJMYW5kIHVzZSBwYXRjaGVzIiwiRnVuY3Rpb25hbCBkaXN0cmljdCBwYXRjaGVzIikNCmxmdHlwZTE8LXJlcChsZnR5cGUxLGVhY2g9MzAwKQ0KTENVMzA8LWRhdGEuZnJhbWUoc2l6ZT1MQ1UkVjRbYygxOjYwMCldLGxhY3VuYXJpdHk9TENVJFY2W2MoMTo2MDApXSx0eXBlPWxmdHlwZTEpDQoNCmxmdHlwZTI8LWMoIkxhbmQgdXNlIHBhdGNoZXMiLCJGdW5jdGlvbmFsIGRpc3RyaWN0IHBhdGNoZXMiKQ0KbGZ0eXBlMjwtcmVwKGxmdHlwZTIsZWFjaD0yMCkNCkxDVTUwMDwtZGF0YS5mcmFtZShzaXplPUxDVSRWNFtjKDYwMTo2NDApXSxsYWN1bmFyaXR5PUxDVSRWNltjKDYwMTo2NDApXSx0eXBlPWxmdHlwZTIpDQoNCmdndGhlbXIoImZyZXNoIikNCg0KTENVcGxvdDwtcmJpbmQoTENVMzAsTENVNTAwKQ0KDQpjcHgxPC0yLjQ4OQ0KY3B5MTwtLTAuMjU5NzI0NCpjcHgxKzAuMDYxMTE3NypjcHgxXjItMC4wMDgxODU2KmNweDFeMysxLjE3OTcxODYNCmNweDI8LTMuMDgwDQpjcHkyPC0tMC40MjIzNTc3KmNweDIrMC4wOTQzNTA4KmNweDJeMi0wLjAxMDIxMTQqY3B4Ml4zKzEuNDA1OTM1NQ0KY3B4MzwtbG9nKExDVXBsb3RbNjAyLDFdKQ0KY3B5MzwtbG9nKExDVXBsb3RbNjAyLDJdKQ0KY3B4NDwtbG9nKExDVXBsb3RbNjIyLDFdKQ0KY3B5NDwtbG9nKExDVXBsb3RbNjIyLDJdKQ0KY3B4MTwtcmVwKGNweDEsMzAwKQ0KY3B4MjwtcmVwKGNweDIsMzAwKQ0KY3B4MzwtcmVwKGNweDMsMjApDQpjcHg0PC1yZXAoY3B4NCwyMCkNCmNweDwtYyhjcHgxLGNweDIsY3B4MyxjcHg0KQ0KY3B5PC1jKGNweTEsY3B5MixjcHkzLGNweTQpDQpjcHR5cGU8LWMoIkxhbmQgdXNlIHBhdGNoZXMiLCJGdW5jdGlvbmFsIGRpc3RyaWN0IHBhdGNoZXMiKQ0KY3B0eXBlPC1yZXAoY3B0eXBlLDIpDQpjcHNjYWxlPC1jKCIoYSkiLCIoYikiKQ0KY3BzY2FsZTwtcmVwKGNwc2NhbGUsMikNCmNweHk8LWRhdGEuZnJhbWUoY3B4LGNweSkNCmNweGxpbmU8LWRhdGEuZnJhbWUoY3B4LHR5cGU9Y3B0eXBlLHNjYWxlPWNwc2NhbGUpDQoNCkxDVXBsb3Q8LXJiaW5kKExDVTMwLExDVTUwMCkNCnNjYWxlTENVPC1jKCIoYSkiLCIoYikiKQ0Kc2NhbGVMQ1U8LXJlcChzY2FsZUxDVSxlYWNoPTYwMCkNCnNjYWxlTENVPC1zY2FsZUxDVVsxOjY0MF0NCkxDVXBsb3Q8LWRhdGEuZnJhbWUoTENVcGxvdCxzY2FsZT1zY2FsZUxDVSkNCkxDVXBsb3Q8LWRhdGEuZnJhbWUoTENVcGxvdCxzY2FsZT1zY2FsZUxDVSxjcHh5KQ0KDQpwMzA8LWdncGxvdChzdWJzZXQoTENVMzAsIExDVTMwJHR5cGU9PSJGdW5jdGlvbmFsIGRpc3RyaWN0IHBhdGNoZXMiKSkrZ2VvbV9wb2ludChtYXBwaW5nPWFlcyh4PWxvZyhzaXplKSx5PWxvZyhsYWN1bmFyaXR5KSxjb2xvdXI9dHlwZSxzaGFwZT10eXBlKSkrDQogIGdlb21fbGluZShtYXBwaW5nPWFlcyh4PWxvZyhzaXplKSx5PWxvZyhsYWN1bmFyaXR5KSxjb2xvdXI9dHlwZSkpKw0KICBnZW9tX3ZsaW5lKHhpbnRlcmNlcHQ9Y3B4WzE6Ml0sY29sb3I9InJlZCIsbGluZXR5cGU9ImRhc2hlZCIpKw0KICB5bGltKDAsMS41KStsYWJzKHg9ImxuKGJveCBzaXplKS9jZWxsIix5PSJsbihMYWN1bmFyaXR5KSIsdGl0bGU9IihhKSIpKw0KICB0aGVtZShsZWdlbmQucG9zaXRpb249Ik5vbmUiLHRpdGxlPWVsZW1lbnRfdGV4dChzaXplPTI1LGZhbWlseT0iUlQiKSxheGlzLnRleHQueT1lbGVtZW50X3RleHQoc2l6ZT0yNSxmYW1pbHk9IlJUIiksDQogICAgICAgIGF4aXMudGV4dC54PWVsZW1lbnRfdGV4dChzaXplPTI1LGZhbWlseT0iUlQiKSxheGlzLnRpdGxlPWVsZW1lbnRfdGV4dChzaXplPTI1LGZhbWlseT0iUlQiKSwNCiAgICAgICAgc3RyaXAudGV4dD1lbGVtZW50X3RleHQoc2l6ZT0yNSxmYW1pbHk9IlJUIikpDQoNCnA1MDA8LWdncGxvdChzdWJzZXQoTENVNTAwLCBMQ1U1MDAkdHlwZT09IkZ1bmN0aW9uYWwgZGlzdHJpY3QgcGF0Y2hlcyIpKStnZW9tX3BvaW50KG1hcHBpbmc9YWVzKHg9bG9nKHNpemUpLHk9bG9nKGxhY3VuYXJpdHkpLGNvbG91cj10eXBlLHNoYXBlPXR5cGUpKSsNCiAgZ2VvbV9saW5lKG1hcHBpbmc9YWVzKHg9bG9nKHNpemUpLHk9bG9nKGxhY3VuYXJpdHkpLGNvbG91cj10eXBlKSkrDQogIGdlb21fdmxpbmUoeGludGVyY2VwdD1jcHhbMzo0XSxjb2xvcj0icmVkIixsaW5ldHlwZT0iZGFzaGVkIikrDQogIHlsaW0oMCwwLjc1KStsYWJzKHg9ImxuKGJveCBzaXplKS9jZWxsIix5PSJsbihMYWN1bmFyaXR5KSIsdGl0bGU9IihiKSIpKw0KICB0aGVtZShsZWdlbmQucG9zaXRpb249Ik51bGwiLGxlZ2VuZC50aXRsZT1lbGVtZW50X3RleHQoc2l6ZT0yNSxmYW1pbHk9IlJUIiksDQogICAgICAgIGxlZ2VuZC50ZXh0PWVsZW1lbnRfdGV4dChzaXplPTI1LGZhbWlseT0iUlQiKSxsZWdlbmQua2V5LmhlaWdodD11bml0KDEsImNtIiksDQogICAgICAgIHRpdGxlPWVsZW1lbnRfdGV4dChzaXplPTI1LGZhbWlseT0iUlQiKSxheGlzLnRleHQueT1lbGVtZW50X3RleHQoc2l6ZT0yNSxmYW1pbHk9IlJUIiksDQogICAgICAgIGF4aXMudGV4dC54PWVsZW1lbnRfdGV4dChzaXplPTI1LGZhbWlseT0iUlQiKSxheGlzLnRpdGxlPWVsZW1lbnRfdGV4dChzaXplPTI1LGZhbWlseT0iUlQiKSwNCiAgICAgICAgc3RyaXAudGV4dD1lbGVtZW50X3RleHQoc2l6ZT0yNSxmYW1pbHk9IlJUIikpDQpzY2FsZXBsb3Q8LWdyaWQuYXJyYW5nZShwMzAscDUwMCxucm93PTEpDQpgYGANCg0KDQpEb3dubG9hZCBsaW5rcyBhcyBmb2xsb3c6DQoNCltSIDMwIG1dKGh0dHA6Ly9zY2llbmNlLmdpc2Vyc3FkYWkudG9wL0RJQi9MQ1UzMC50eHQpDQpbUiA1MDAgbV0oaHR0cDovL3NjaWVuY2UuZ2lzZXJzcWRhaS50b3AvRElCL0xDVTUwMC50eHQpDQoNCiMjIExhbmRzY2FwZSBtZXRyaWNzDQpGcmFnc3RhdHMgNC4yIHNvZnR3YXJlIHdhcyB1c2VkIHRvIGNhbGN1bGF0ZSB0aGUgbnVtYmVyIG9mIHBhdGNoZXMgKE5QKSwgcGF0Y2ggZGVuc2l0eSAoUEQpLCBkaXZpc2lvbiAoRElWSVNJT04pIGFuZCBlZmZlY3RpdmUgbWVzaCBzaXplIChNRVNIKSBtZXRyaWNzLg0KDQojIyMgMzAgbSByZXNvbHV0aW9uIG1hcHMNCmBgYHtyIGVjaG89RkFMU0UsIG1lc3NhZ2U9RkFMU0UsIHdhcm5pbmc9RkFMU0V9DQpOUDMwIDwtICByYXN0ZXIoIkY6L0dJU3Byb2plY3QvSVVFL0NhcmJvbkVtaXNzaW9uL0ZpbmFsL1NlY29uZFJlc3VsdC9FY29sb2dpY2FsSW5kZXgvbXVsdGlwbGVsYW5kc2NhcGUvcGxhbm5pbmdmdW5jdGlvbjMwL25wLnRpZiIpDQpQRDMwIDwtIHJhc3RlcigiRjovR0lTcHJvamVjdC9JVUUvQ2FyYm9uRW1pc3Npb24vRmluYWwvU2Vjb25kUmVzdWx0L0Vjb2xvZ2ljYWxJbmRleC9tdWx0aXBsZWxhbmRzY2FwZS9wbGFubmluZ2Z1bmN0aW9uMzAvcGQudGlmIikNCkRJVklTSU9OMzAgPC0gcmFzdGVyKCJGOi9HSVNwcm9qZWN0L0lVRS9DYXJib25FbWlzc2lvbi9GaW5hbC9TZWNvbmRSZXN1bHQvRWNvbG9naWNhbEluZGV4L211bHRpcGxlbGFuZHNjYXBlL3BsYW5uaW5nZnVuY3Rpb24zMC9kaXZpc2lvbi50aWYiKQ0KTUVTSDMwIDwtIHJhc3RlcigiRjovR0lTcHJvamVjdC9JVUUvQ2FyYm9uRW1pc3Npb24vRmluYWwvU2Vjb25kUmVzdWx0L0Vjb2xvZ2ljYWxJbmRleC9tdWx0aXBsZWxhbmRzY2FwZS9wbGFubmluZ2Z1bmN0aW9uMzAvbWVzaC50aWYiKQ0KDQptMTEgPC0gbWFwdmlldyhOUDMwLCBsYXllci5uYW1lID0gIk5QIikNCm0xMiA8LSBtYXB2aWV3KFBEMzAsIGxheWVyLm5hbWUgPSAiUEQiKQ0KbTEzIDwtIG1hcHZpZXcoRElWSVNJT04zMCwgbGF5ZXIubmFtZSA9ICJESVZJU0lPTiIpDQptMTQgPC0gbWFwdmlldyhNRVNIMzAsIGxheWVyLm5hbWUgPSAiTUVTSCIpDQpzeW5jKG0xMSwgbTEyLCBtMTMsIG0xNCkNCmBgYA0KDQpEb3dubG9hZCBsaW5rcyBhcyBmb2xsb3c6DQoNCltOUF0oaHR0cDovL3NjaWVuY2UuZ2lzZXJzcWRhaS50b3AvRElCL25wMzAudGlmKQ0KDQpbUERdKGh0dHA6Ly9zY2llbmNlLmdpc2Vyc3FkYWkudG9wL0RJQi9wZDMwLnRpZikNCg0KW0RJVklTSU9OXShodHRwOi8vc2NpZW5jZS5naXNlcnNxZGFpLnRvcC9ESUIvZGl2aXNpb24zMC50aWYpDQoNCltNRVNIXShodHRwOi8vc2NpZW5jZS5naXNlcnNxZGFpLnRvcC9ESUIvbWVzaDMwLnRpZikNCg0KIyMjIDUwMCBtIHJlc29sdXRpb24gbWFwcw0KYGBge3IgZWNobz1GQUxTRSwgbWVzc2FnZT1GQUxTRSwgd2FybmluZz1GQUxTRX0NCk5QNTAwIDwtICByYXN0ZXIoIkY6L0dJU3Byb2plY3QvSVVFL0NhcmJvbkVtaXNzaW9uL0ZpbmFsL1NlY29uZFJlc3VsdC9FY29sb2dpY2FsSW5kZXgvbXVsdGlwbGVsYW5kc2NhcGUvZnVuY3Rpb241MDAvbnAudGlmIikNClBENTAwIDwtIHJhc3RlcigiRjovR0lTcHJvamVjdC9JVUUvQ2FyYm9uRW1pc3Npb24vRmluYWwvU2Vjb25kUmVzdWx0L0Vjb2xvZ2ljYWxJbmRleC9tdWx0aXBsZWxhbmRzY2FwZS9mdW5jdGlvbjUwMC9wZC50aWYiKQ0KRElWSVNJT041MDAgPC0gcmFzdGVyKCJGOi9HSVNwcm9qZWN0L0lVRS9DYXJib25FbWlzc2lvbi9GaW5hbC9TZWNvbmRSZXN1bHQvRWNvbG9naWNhbEluZGV4L211bHRpcGxlbGFuZHNjYXBlL2Z1bmN0aW9uNTAwL2RpdmlzaW9uLnRpZiIpDQpNRVNINTAwIDwtIHJhc3RlcigiRjovR0lTcHJvamVjdC9JVUUvQ2FyYm9uRW1pc3Npb24vRmluYWwvU2Vjb25kUmVzdWx0L0Vjb2xvZ2ljYWxJbmRleC9tdWx0aXBsZWxhbmRzY2FwZS9mdW5jdGlvbjUwMC9tZXNoLnRpZiIpDQoNCm0xNSA8LSBtYXB2aWV3KE5QNTAwLCBsYXllci5uYW1lID0gIk5QIikNCm0xNiA8LSBtYXB2aWV3KFBENTAwLCBsYXllci5uYW1lID0gIlBEIikNCm0xNyA8LSBtYXB2aWV3KERJVklTSU9ONTAwLCBsYXllci5uYW1lID0gIkRJVklTSU9OIikNCm0xOCA8LSBtYXB2aWV3KE1FU0g1MDAsIGxheWVyLm5hbWUgPSAiTUVTSCIpDQpzeW5jKG0xNSwgbTE2LCBtMTcsIG0xOCkNCmBgYA0KDQpEb3dubG9hZCBsaW5rcyBhcyBmb2xsb3c6DQoNCltOUF0oaHR0cDovL3NjaWVuY2UuZ2lzZXJzcWRhaS50b3AvRElCL25wNTAwLnRpZikNCg0KW1BEXShodHRwOi8vc2NpZW5jZS5naXNlcnNxZGFpLnRvcC9ESUIvcGQ1MDAudGlmKQ0KDQpbRElWSVNJT05dKGh0dHA6Ly9zY2llbmNlLmdpc2Vyc3FkYWkudG9wL0RJQi9kaXZpc2lvbjUwMC50aWYpDQoNCltNRVNIXShodHRwOi8vc2NpZW5jZS5naXNlcnNxZGFpLnRvcC9ESUIvbWVzaDUwMC50aWYpDQoNCiMjIEltcGFjdCBmYWN0b3JzIG9mIHRoZSAkQ09fMiQgbWl0aWdhdGlvbjogUFVBIGFuZCBQT0lEDQoNCiMjIyBQVUENCg0KYGBge3IgZWNobz1GQUxTRSwgbWVzc2FnZT1GQUxTRSwgd2FybmluZz1GQUxTRX0NClBVQTMwIDwtIHJhc3RlcigiRjovR0lTcHJvamVjdC9JVUUvQ2FyYm9uRW1pc3Npb24vRmluYWwvU2Vjb25kUmVzdWx0L0ludGVyYWN0aW9uL1VyYmFuQXJlYS9QVUEzMC50aWYiKQ0KUFVBNTAwIDwtIHJhc3RlcigiRjovR0lTcHJvamVjdC9JVUUvQ2FyYm9uRW1pc3Npb24vRmluYWwvU2Vjb25kUmVzdWx0L0ludGVyYWN0aW9uL1VyYmFuQXJlYS9QVUE1MDAudGlmIikNCg0KbTE5IDwtIG1hcHZpZXcoUFVBMzAqMTAwLCBsYXllci5uYW1lID0gIlIgMzAgbS9QZXJjZW50YWdlIikNCm0yMCA8LSBtYXB2aWV3KFBVQTUwMCoxMDAsIGxheWVyLm5hbWUgPSAiUiA1MDAgbS9QZXJjZW50YWdlIikNCg0Kc3luYyhtMTksIG0yMCkNCmBgYA0KDQpEb3dubG9hZCBsaW5rcyBhcyBmb2xsb3c6DQoNCltSIDMwIG1dKGh0dHA6Ly9zY2llbmNlLmdpc2Vyc3FkYWkudG9wL0RJQi9QVUEzMC50aWYpDQoNCltSIDUwMCBtXShodHRwOi8vc2NpZW5jZS5naXNlcnNxZGFpLnRvcC9ESUIvUFVBNTAwLnRpZikNCg0KIyMjIFBPSUQNCg0KYGBge3IgZWNobz1GQUxTRSwgbWVzc2FnZT1GQUxTRSwgd2FybmluZz1GQUxTRX0NClBPSUQzMCA8LSByYXN0ZXIoIkY6L0dJU3Byb2plY3QvSVVFL0NhcmJvbkVtaXNzaW9uL0ZpbmFsL1NlY29uZFJlc3VsdC9kYXRhc2hhcmV1c2UvUE9JRDMwLnRpZiIpDQpQT0lENTAwIDwtIHJhc3RlcigiRjovR0lTcHJvamVjdC9JVUUvQ2FyYm9uRW1pc3Npb24vRmluYWwvU2Vjb25kUmVzdWx0L2RhdGFzaGFyZXVzZS9QT0lENTAwLnRpZiIpDQoNCm0yMSA8LSBtYXB2aWV3KFBPSUQzMCwgbGF5ZXIubmFtZSA9ICJSIDMwIG0iKQ0KbTIyIDwtIG1hcHZpZXcoUE9JRDUwMCwgbGF5ZXIubmFtZSA9ICJSIDUwMCBtIikNCg0Kc3luYyhtMjEsIG0yMikNCmBgYA0KDQoNCkRvd25sb2FkIGxpbmtzIGFzIGZvbGxvdzoNCg0KW1IgMzAgbV0oaHR0cDovL3NjaWVuY2UuZ2lzZXJzcWRhaS50b3AvRElCL1BPSUQzMC50aWYpDQoNCltSIDUwMCBtXShodHRwOi8vc2NpZW5jZS5naXNlcnNxZGFpLnRvcC9ESUIvUE9JRDUwMC50aWYpDQoNCiMgUmVmZXJlbmNlIA0KSWYgeW91IHVzZWQgdGhlIGRhdGFbQFJOOTEyOyBAWlVPMjAxOTExODY1OV0sIHBsZWFzZSBjaXRlZCBhcyBmb2xsb3c6DQo=
