# Supplementary figures and images for: A spatial database of CO2 emissions, urban form fragmentation and city-scale effect related impact factors for the low carbon urban system in Jinjiang city, China
Source: Data Brief. 2020 Feb 11;29:105274. doi: 10.1016/j.dib.2020.105274 (PMC7042417; doi:10.1016/j.dib.2020.105274)

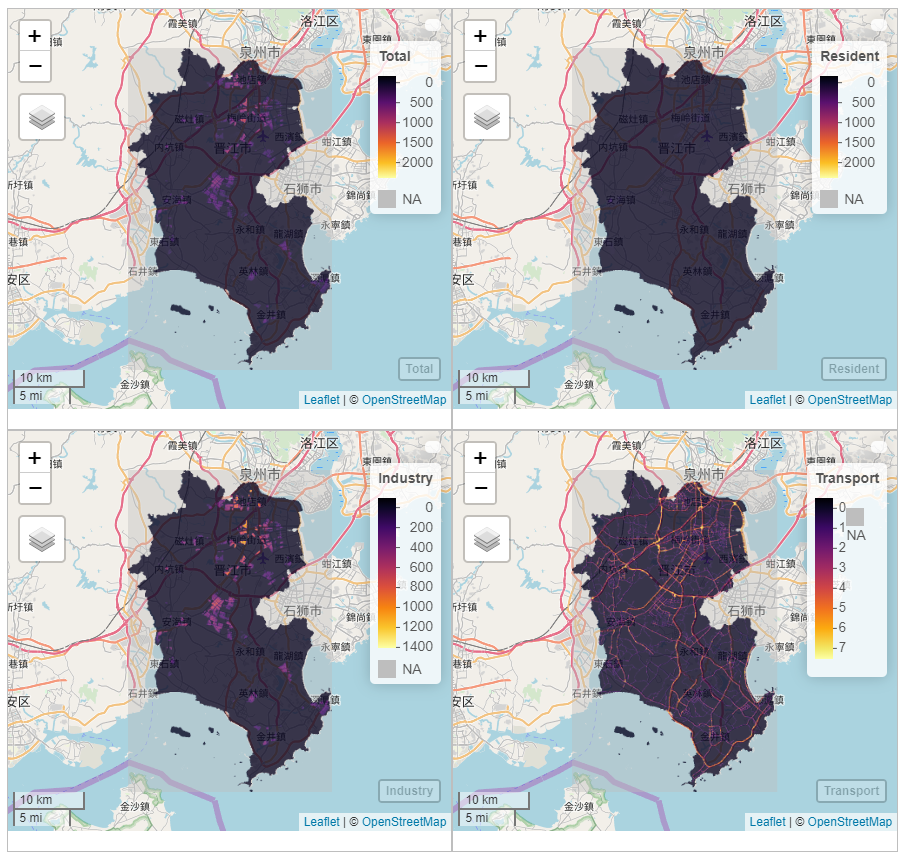

Supplement: Multimedia component 1 [file mmc1.zip › 数据包/30m.png]

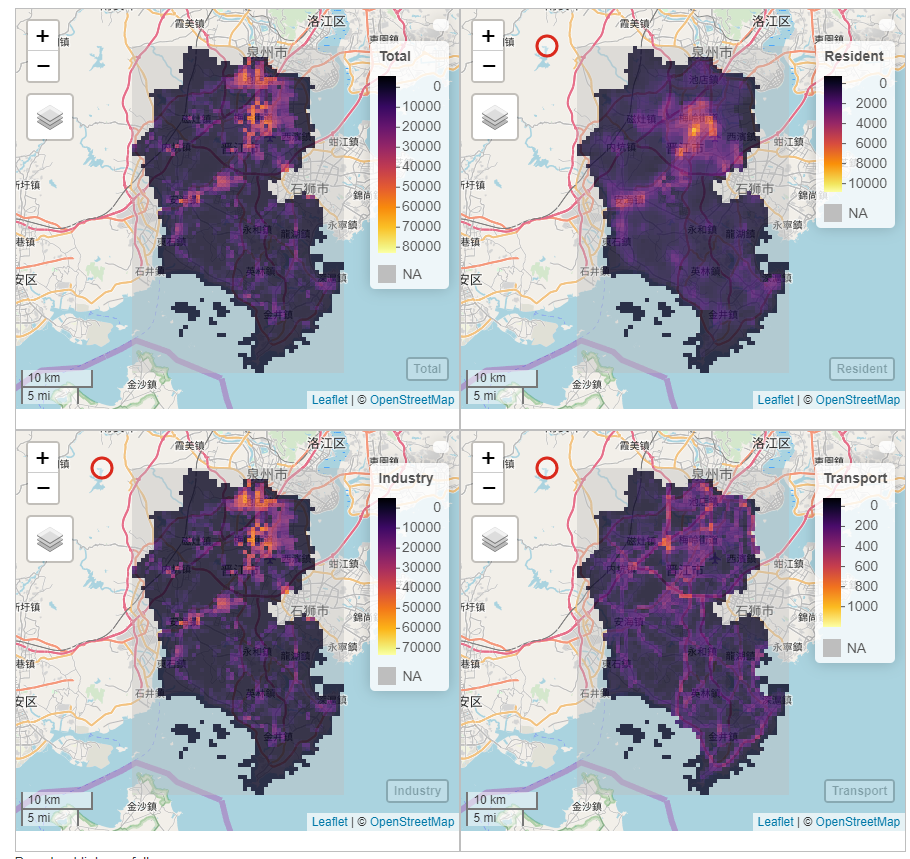

Supplement: Multimedia component 1 [file mmc1.zip › 数据包/500m.png]

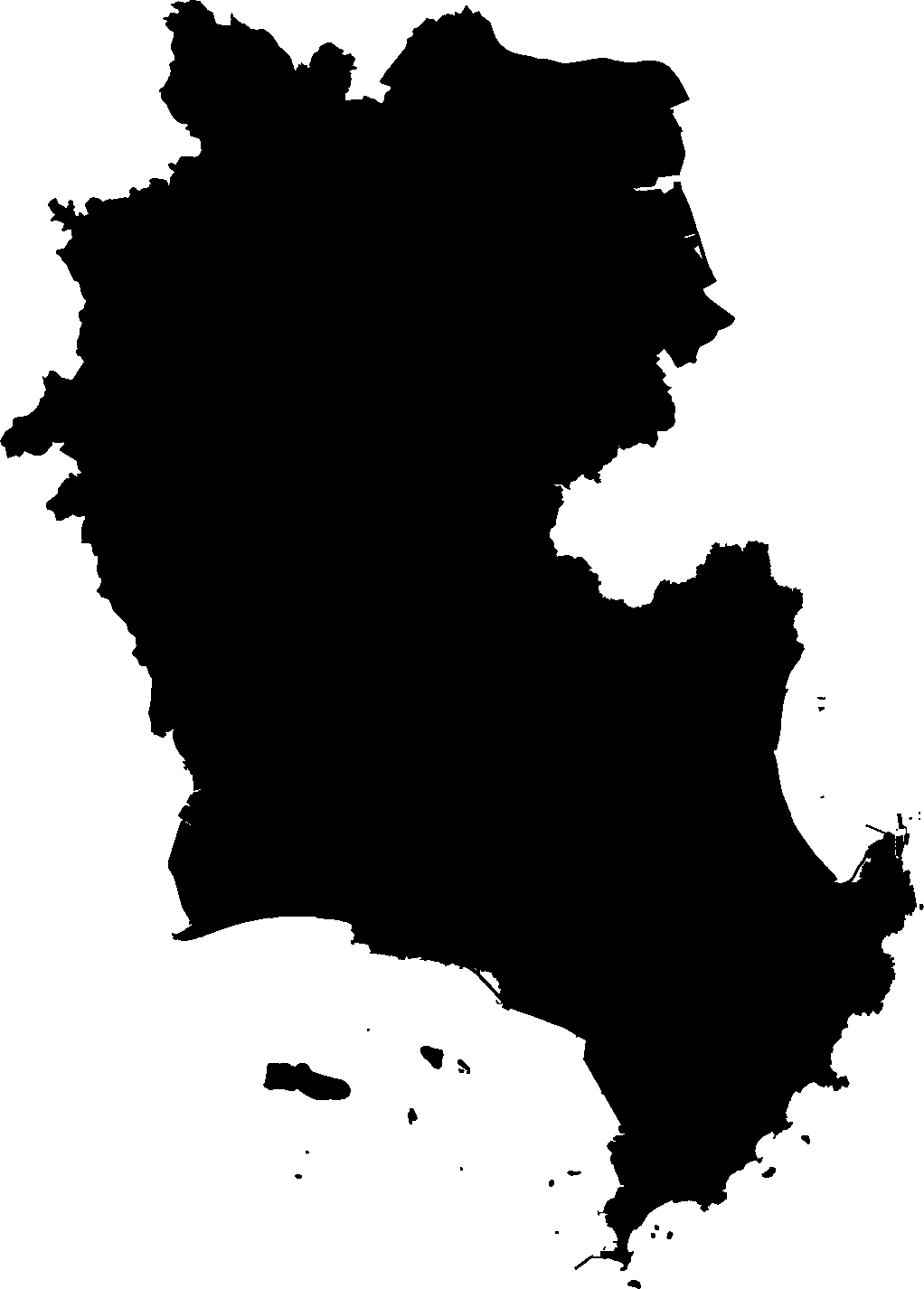

Supplement: Multimedia component 1 [file mmc1.zip › 数据包/dataset/POID30.tif]

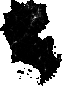

Supplement: Multimedia component 1 [file mmc1.zip › 数据包/dataset/POID500.tif]
